# Efficacy and safety of salvianolate injection in treating acute myocardial infarction: a meta-analysis and systematic literature review

**DOI:** 10.3389/fphar.2024.1478558

**Published:** 2024-12-17

**Authors:** Pengfei Chen, He Zhang, Zhuye Gao, Dazhuo Shi, Jie Zhang

**Affiliations:** ^1^ Xiyuan Hospital, China Academy of Chinese Medical Sciences, Beijing, China; ^2^ Cardiovascular Diseases Center, Xiyuan Hospital, China Academy of Chinese Medical Sciences, Beijing, China

**Keywords:** salvianolic acids, salvianolic acid B, acute myocardial infarction, randomized controlled trials, systematic review

## Abstract

**Purpose:**

Salvianolate for injection (SFI) is a widely used treatment for acute myocardial infarction (AMI). This study aims to assess the efficacy and safety of SFI in treating AMI by synthesizing evidence from published randomized controlled trials (RCTs).

**Methods:**

Seven databases were searched for relevant RCTs published up to 1 July 2024. Two investigators independently conducted the literature searches, data extraction, and quality assessment. Subgroup and sensitivity analyses were performed to address potential heterogeneity. Data analyses were conducted using RevMan 5.4 software.

**Result:**

Thirty RCTs with a total of 3,931 participants were included in the study and analyzed. The results revealed that SFI significantly reduced major adverse cardiac events (MACEs) (RR = 0.34, 95% CI: 0.24 to 0.49, *p* < 0.05). In addition, SFI lowered creatine kinase-MB (CK-MB) (MD = −5.65, 95% CI: −9.55 to −1.76, *p* < 0.05) and improved left ventricular ejection fraction (LVEF) (MD = 6.2, 95% CI: 4.82 to 7.57, *p* < 0.05). Further reductions were observed in C-reactive protein (CRP) (MD = −6.17, 95% CI: −8.11 to −4.23, *p* < 0.05), malondialdehyde (MDA) (MD = −1.95, 95% CI: −2.08 to −1.83, *p* < 0.05), and endothelin-1 (ET-1) (MD = −12.27, 95% CI: −17.13 to −7.40, *p* < 0.05). The incidence of adverse events did not significantly differ between the EG and CG [RR = 0.74, 95% CI: 0.42 to 1.33, *p* = 0.32].

**Conclusion:**

This study suggests that SFI may be a promising alternative therapy for treating AMI without increasing the risk of adverse events. However, our findings may be limited by the quality of the existing studies. High-quality RCTs are needed to provide more robust evidence.

**Systematic Review Registration:**

https://www.crd.york.ac.uk/PROSPERO/, identifier CRD42024567279.

## Introduction

Acute myocardial infarction (AMI) is a critical cardiovascular condition characterized by the sudden obstruction of coronary arteries, typically resulting from atherosclerotic plaque rupture or thrombus formation, leading to myocardial cell necrosis (Reed et al., 2017). Globally, AMI is responsible for 9.14 million fatalities, constituting 49.2% of all cardiovascular disease (CVD)-related deaths (Nichols et al., 2014; Yeh et al., 2010). The immediate management of AMI prioritizes the rapid restoration of blood flow to the occluded coronary artery to achieve myocardial reperfusion, with percutaneous coronary intervention (PCI) being the preferred therapeutic approach (Gunnar et al., 1990; De Luca et al., 2008). Concurrently, a combination of antiplatelet agents, anticoagulants, vasodilators, and other supportive medications is administered to maintain myocardial function and mitigate further damage (Windecker et al., 2013; Levine et al., 2016; Puymirat et al., 2017; Valgimigli and Gragnano, 2020). Although these interventions effectively reduce myocardial injury in the acute phase, reperfusion injury may occur, potentially causing additional damage to cardiomyocytes during the restoration of blood flow (Puymirat et al., 2017). Moreover, current treatment strategies predominantly focus on acute management, inadequately addressing the long-term structural and functional recovery of the heart. Consequently, AMI patients continue to face elevated morbidity and mortality rates in the long term.

Danshen, scientifically known as Salvia miltiorrhiza Bunge, is a traditional Chinese medicinal botanical drug utilized in the treatment of various conditions, including myocardial infarction, ischemic stroke, and hepatitis (Ho and Hong, 2011; Xu et al., 2018). Salvianolate for injection (SFI), a metabolite derived from Danshen, received approval from the Chinese Food and Drug Administration in 2005 for the treatment of CVD (Cao et al., 2021; Zhang et al., 2016). This formulation primarily comprises salvianolic acid B (Sal-B) and its homologous metabolites (Li W. et al., 2018; Hu et al., 2024). SFI has been reported to confer cardiovascular benefits through multiple mechanisms, including anti-inflammatory, anti-apoptotic, anti-ischemia reperfusion injury, and anti-fibrotic effects (Liang et al., 2021; Xu et al., 2023; Qiu et al., 2018; Jiang et al., 2023; Ma et al., 2019). In China, SFI has been employed in clinical settings as an adjunctive therapy for AMI. However, a comprehensive literature review evaluating its efficacy and safety has not been conducted. Therefore, this study aims to elucidate the cardioprotective effects of SFI in patients with AMI through a systematic review of existing research.

## Methods

The protocol were registered in the PROSPERO, with registration number CRD42024567279. This study was carried out following the protocol and in compliance with the PRISMA 2020 guidelines.

### Inclusion criteria

Studies were included according to the four criteria:

(1) Study type: randomized controlled trials (RCTs) assessing the efficacy and safety of SFI for AMI, with no status, language, or data restrictions;
(2) Participants: patients diagnosed with AMI according to the diagnostic criteria outlined in the 2023 European Society of Cardiology (ESC) guidelines (Byrne et al., 2023), the Fourth Universal Definition of Myocardial Infarction (2018) (Thygesen et al., 2018), or the American College of Cardiology (ACC)/American Heart Association (AHA) guidelines (O'Gara et al., 2013). These criteria include typical ischemic symptoms, electrocardiographic changes indicative of ischemia (such as ST-segment elevation or new left bundle branch block), and elevated cardiac troponin levels with a rise and/or fall indicative of myocardial injury;
(3) Interventions: the experimental groups (EG) received both SFI and conventional therapy (CT), while control groups (CG) received only CT. The courses and dosages of SFI were not restricted;
(4) Outcomes: the primary efficacy outcome was evaluated by major adverse cardiac events (MACEs). The secondary outcomes included myocardial injury markers, such as creatine kinase-MB (CK-MB), cardiac troponin I (cTnI), and lactate dehydrogenase (LDH); cardiac function indices, including N-terminal pro-B-type natriuretic peptide (NT-proBNP), left ventricular ejection fraction (LVEF), left ventricular end-diastolic diameter (LVEDD), left ventricular end-diastolic volume (LVEDV), and left ventricular end-systolic volume (LVESV). Inflammatory markers included C-reactive protein (CRP), tumor necrosis factor-alpha (TNF-α), interleukin-6 (IL-6), and toll-like receptor-4 (TLR-4). Oxidative stress markers were malondialdehyde (MDA) and superoxide dismutase (SOD). Vascular endothelial function was assessed using endothelin-1 (ET-1) and nitric oxide (NO). Hemorheological indicators included whole blood specific viscosity (WBSV), plasma specific viscosity (PSV), and fibrinogen. Platelet function was evaluated by measuring P-selectin (CD62p) and CD63. Safety was assessed by adverse events.

### Exclusion criteria

Studies were excluded according to the four criteria:

(1) Unclear reporting of interventions;
(2) Insufficient data for statistical analysis;
(3) Duplicate publications or data;
(4) Conference abstracts, reviews, and technical reports.

### Search strategy

The keywords used were “salvianolate,” “salvianolic acids,” “danshen polyphenolate salts,” “acute myocardial infarction,” and “randomized controlled trial” in both English and Chinese. Literature search was carried out independently by two investigators (PFC and HZ) in seven databases, including PubMed, Web of Science, EMBASE, Cochrane Library, CNKI, VIP, and Wanfang. The search period spanned from each database’s inception to 1 July 2024. Additionally, references from similar systematic reviews were manually checked to ensure all relevant studies were included. Detailed search strategies and screening processes are provided in Supplementary Table S1.

### Study selection and data extraction

Two investigators (PFC and HZ) independently screened studies by examining titles, abstracts, and full texts according to the eligibility criteria. Any disputes were settled by the senior reviewer (DZS).

The following information was extracted, including trial characteristics (first author, publication year, country, follow-up period); patient characteristics (sample size, gender, age); SFI treatments (start time, administration frequency, dosage, duration); and treatment outcomes.

### Bias risk

Two investigators (PFC and HZ) independently assessed the quality and risk of bias using the RoB 2 tool proposed by Cochrane (Sterne et al., 2019). The tool used algorithms to assign responses to signaling questions and generate a risk-of-bias judgment. We assessed five domains: the randomization process, deviations from intended interventions, missing outcome data, outcome measurement, and selective reporting. Each was categorized as either “low risk,” “some concerns,” or “high risk.”

### Statistical analysis

In this study, data analysis was performed using the RevMan 5.4. Continuous variables were expressed as weighted mean differences (WMD) and 95% confidence intervals (CIs), while dichotomous variables were presented as pooled risk ratios (RR) and 95% CIs. Heterogeneity among the RCTs was evaluated using the Cochrane’s Q test and *I* ^*2*^ statistic. Significant statistical heterogeneity was indicated by *p* < 0.05 or *I* ^*2*^ > 50%, in which case a random-effects model was employed to assess outcomes; otherwise, a fixed-effects model was applied. For analyses involving only two trials, a random-effects model was chosen regardless of heterogeneity significance to ensure result accuracy. A p-value of less than 0.05 denoted a statistically significant difference.

### Subgroup and sensitivity analysis

Subgroup and sensitivity analyses were performed to identify the sources of heterogeneity when *I* ^*2*^ was ≥50%. Subgroup analysis focused on treatment duration and follow-up time. Sensitivity analysis involved omitting one study at a time to identify sources of heterogeneity related to sample, gender, age, and interventions.

### Publication bias

For datasets with 10 or more trials, a funnel plot was used to assess publication bias. While we planned to use Egger’s or Begg’s test, these tests were deemed unreliable for datasets with fewer than 10 trials, and thus were not performed.

## Results

### Study selection

**FIGURE 1:**
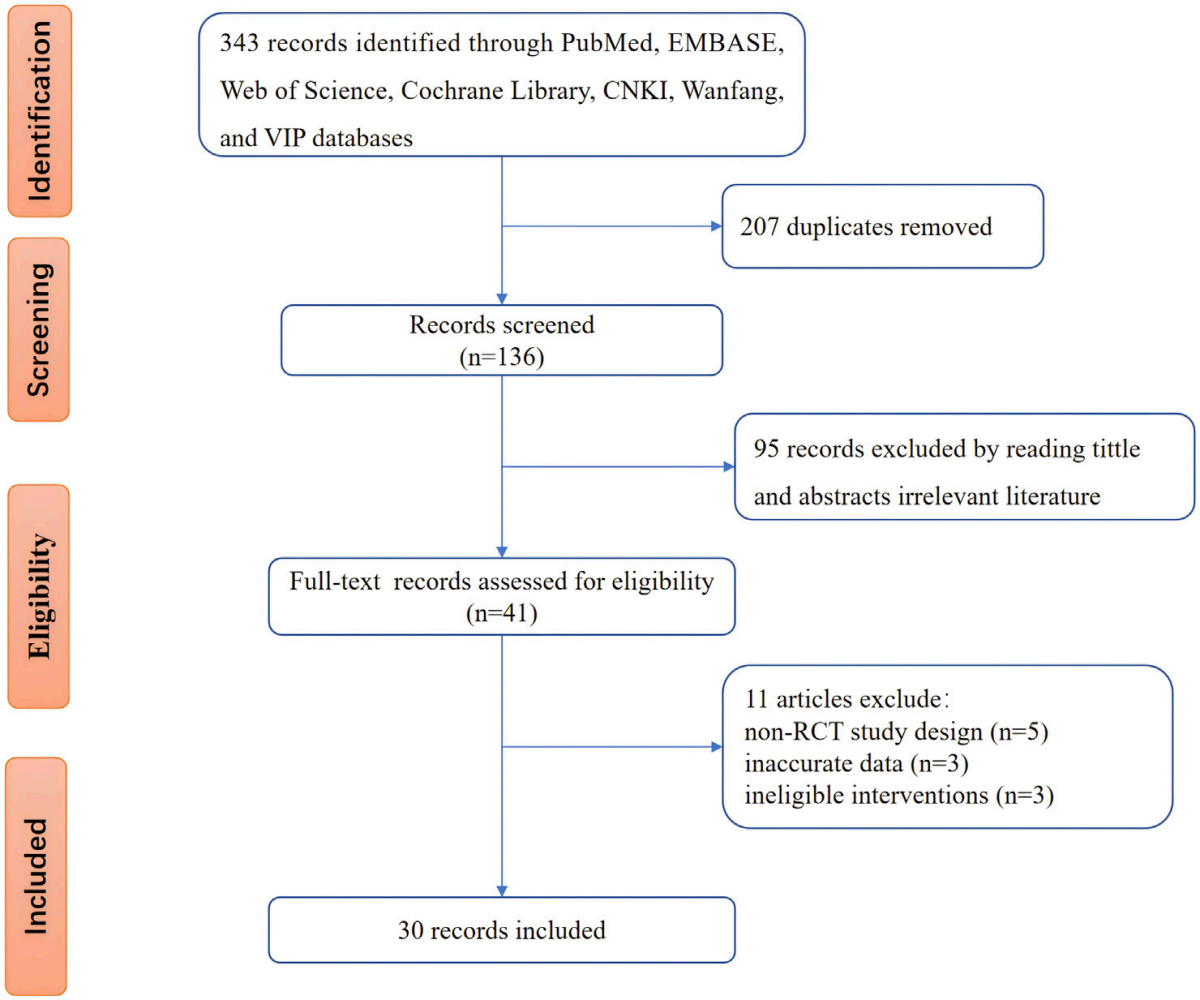
Flow chart of study selection and identification.

Figure 1 illustrates the database search process and study identification. Initially, 343 potentially relevant articles were identified (PubMed: 35, Embase: 42, Web of Science: 38, Cochrane Library: 9, CNKI: 85, Wanfang: 76, VIP: 40, other sources: 18). After excluding 207 duplicate records and 95 ineligible records through title and abstract screening, 41 articles underwent full-text review. Of these, 11 were excluded due to non-RCT study design, inaccurate data, or ineligible interventions. The excluded studies and reasons are detailed in Supplementary Table S2. Ultimately, 30 studies (Shen L. et al., 2020; Ou et al., 2020; Wang, 2018; Wang et al., 2017; Yi et al., 2017; Zu-shan et al., 2016; Wu et al., 2016; Wang et al., 2013; Ye et al., 2014; Qiu, 2019; Zhu and Wang, 2018; Tang, 2023; Liu et al., 2020; Liu et al., 2022; Zhang et al., 2022; Zhang et al., 2017; Hu Xiaochun et al., 2023; Hongmei et al., 2017; Jizheng and Lu, 2020; Guo, 2017; Duan and Jian-Hong, 2016; Dong et al., 2019; He et al., 2014; Hou and Yunfeng, 2018; Li et al., 2020; Zhu and Wang, 2022; Fu, 2024; Zhao, 2021; Lan et al., 2011; Lin et al., 2023) were included in our study.

### Characteristics of studies

Table 1 summarizes the characteristics of the 30 RCTs included, involving a total of 3,931 participants (1,972 in the EG and 1,959 in the CG). There were no statistically significant differences in baseline information between the EG and CG. The patients’ ages ranged from 50.38 ± 5.01 to 71.56 ± 8.37 years. The treatment duration with SFI varied from 1 to 21 days. Following the ConPhyMP guidelines (Heinrich et al., 2022), all the included RCTs focused on “type A” extracts, which are listed in the national pharmacopeia and have licensed applications. Detailed information on the source, composition, and chemical properties of SFI used in these trials is provided in Supplementary Table S3.

**TABLE 1:** Characteristics of included studies.

Author (publication year)	Sample size (T/C)	Male/female		Age (year)	Intervention		Courses/ day	Outcomes
		T	C	T/C	T	C		
Dong Yuren (2019)	52/52	32/20	30/22	58.50 ± 3.80/58.60 ± 4.50	Salvianolate 200 mg + 5% G.S. 250 mL, qd, ivgtt	CT	14 day	④⑯⑰⑱⑲㉓
Duan Xinyun (2016)	30/30	17/13	19/11	59.76 ± 7.23/60.00 ± 7.35	Salvianolate 200 mg + 0.9% N.S. 250 mL, qd, ivgtt	CT	—	⑤⑥⑩⑪⑫⑯⑰
Fu Xiaolong (2024)	40/40	22/18	26/14	62.76 ± 3.02/64.02 ± 3.17	Salvianolate 150 mg + 0.9% N.S. 250 mL, qd, ivgtt	CT	14 day	①②④⑥⑦
Guo Xiufang (2017)	58/58	40/18	38/20	63.80 ± 6.40/64.00 ± 6.20	Salvianolate 200 mg + 5% G.S. 250 mL, qd, ivgtt	CT	7 day	②⑩⑭⑮⑯㉑㉒㉓
He Tao (2014)	37/35	—	—	—	Salvianolate 200 mg + 5% G.S. 250 mL, qd, ivgtt	CT	10 day	③⑤⑥⑩⑱⑲⑳㉓
Hou Lifang (2018)	47/46	30/17	27/19	63.64 ± 5.28/62.83 ± 4.92	Salvianolate 150 mg + 0.9% N.S. 250 mL, qd, ivgtt	CT	14 day	①⑥⑦⑧⑨⑭
Hu Xiaochun (2023)	51/51	30/21	28 / 23	51.24 ± 5.42/50.38 ± 5.01	Salvianolate 150 mg + 0.9% N.S. 250 mL, qd, ivgtt	CT	14 day	②④⑤⑥⑧⑨⑭⑮
Li Hongmei (2017)	40/40	26/14	25/15	66.52 ± 4.75/66.75 ± 5.12	Salvianolate 200 mg + 5% G.S. 250 mL, qd, ivgtt	CT	14 day	⑤⑯⑰
Li Jizhong (2020)	49/49	26/23	27/22	62.28 ± 7.63/61.26 ± 7.45	Salvianolate 200 mg + 5% G.S. 250 mL, qd, ivgtt	CT	14 day	①②⑥⑦⑩
Li Sai (2020)	57/57	32/25	33/24	54.14 ± 7.85/53.92 ± 7.92	Salvianolate 200 mg + 5% G.S. 250 mL, qd, ivgtt	CT	14 day	②③⑫
Lin Weibin (2023)	45/45	24/21	25/20	59.12 ± 6.38/58.43 ± 6.56	Salvianolate 200 mg + 0.9% N.S. 250 mL, qd, ivgtt	CT	14 day	②③⑥⑧⑨⑩⑲⑳
Liu Tiezhen (2020)	75/75	45/30	42/33	59.60 ± 3.20/60.30 ± 3.90	Salvianolate 200 mg + 5% G.S. 250 mL, qd, ivgtt	CT	14 day	①⑥⑮⑯㉓
Liu Zhen (2022)	41/41	27/14	25/16	68.61 ± 3.28/68.55 ± 3.05	Salvianolate 200 mg + 0.9% N.S. 250 mL, qd, ivgtt	CT	14 day	⑥⑧⑨
Ni Lan (2011)	80/80	45/35	44/36	71.55 ± 8.35/71.56 ± 8.37	Salvianolate 200 mg + 5% G.S. 250 mL, qd, ivgtt	CT	10 day	③⑤⑩㉓
Qiu Jun (2019)	41/41	28/13	30/11	61.40 ± 6.42/61.33 ± 5.78	Salvianolate 200 mg + 0.9% N.S. 250 mL, qd, ivgtt	CT	—	⑥⑩⑪⑫⑭⑮⑯⑰㉑㉒
Tang Changlin (2023)	45/44	23/21	25/20	52.16 ± 5.39/52.10 ± 5.48	Salvianolate 200 mg + 5% G.S. 250 mL, qd, ivgtt	CT	14 day	⑥⑦⑪
Wang Xifu (2014)	165/155	109/56	113 /42	58.60 ± 11.30/57.30 ± 9.30	Salvianolate 400 mg + 5% G.S. 250 mL, qd, ivgtt	CT	7 day	⑬
Wang Xifu (2017)	150/150	110/40	107/43	60.90 ± 10.30/59.80 ± 10.10	Salvianolate 200 mg + 5% G.S. 250 mL, qd, ivgtt	CT	5 day	⑬
Wang Zerong (2018)	45/45	24/21	25/20	60.80 ± 5.50/60.50 ± 5.30	Salvianolate 200 mg + 5% G.S. 250 mL, qd, ivgtt	CT	7 day	⑤⑥⑩⑬
Wu Dexun (2016)	44/44	29/15	27/17	54.08 ± 3.97/55.97 ± 4.28	Salvianolate 200 mg + 0.9% N.S. 250 mL, qd, ivgtt	CT	7 day	①⑥⑭⑮⑯⑰㉓
Ye Ming (2014)	165/155	109/56	113/42	58.60 ± 11.30/57.30 ± 9.30	Salvianolate 400 mg + 0.9% N.S. 250 mL, qd, ivgtt	CT	7 day	⑭⑮⑯㉑㉒㉓
Yu Zushan (2016)	50/50	26/24	27/23	60.00 ± 4.80/59.00 ± 4.90	Salvianolate 200 mg + 0.9% N.S. 250 mL, qd, ivgtt	CT	7 day	⑩⑮⑰
Zhang Xaiojie (2017)	28/28	16/12	17/11	56.18 ± 9.66/55.72 ± 9.52	Salvianolate 100 mg + 0.9% N.S. 100 mL, bid, ivgtt	CT	14 day	⑥⑦
Zhang Yan (2022)	64/64	36/28	35/29	69.50 ± 5.99/68.94 ± 6.49	Salvianolate 200 mg + 5% G.S. 250 mL, qd, ivgtt	CT	14 day	①⑪⑫⑭⑮
Zhao Jian (2021)	43/43	25/18	23/20	69.04 ± 4.12/69.45 ± 4.20	Salvianolate 200 mg + 5% G.S. 250 mL, qd, ivgtt	CT	21 day	⑥⑦⑨⑩⑪
Zheng Yi (2017)	30/30	17/13	16/14	60.35 ± 8.84/62.23 ± 9.65	Salvianolate 200 mg + 5% G.S. 250 mL, qd, ivgtt	CT	1 day	②③
Zhu Ganlin (2018)	40/40	28/12	25/15	57.05 ± 6.68/56.05 ± 6.50	Salvianolate 200 mg + 0.9% N.S. 100 mL, qd, ivgtt	CT	14 day	⑰
Zhu Ganlin (2021)	38/38	23/15	22/16	68.06 ± 8.86/69.11 ± 9.05	Salvianolate 200 mg + 5% G.S. 250 mL, qd, ivgtt	CT	14 day	①②③⑪⑯⑰
Shen Li (2020)	262/265	219/43	217/48	61.10 ± 12.70/62.40 ± 11.30	Salvianolate 200 mg + 0.9% N.S. 100 mL, qd, ivgtt	CT	7 day	①㉓
Ou Yang (2020)	60/68	54/6	55/13	64.80 ± 11.00/65.20 ± 10.70	Salvianolate 200 mg + 0.9% N.S. 100 mL, qd, ivgtt	CT	3 day	①㉓

T/C, treatment group/control group; CT, conventional treatment; qd, once a day; ivgtt, intravenous guttae; ①MACEs, ②CK-MB, ③cTnI, ④LDH, ⑤NT-proBNP, ⑥LVEF, ⑦LVEDD, ⑧LVEDV, ⑨LVESV, ⑩CRP, ⑪TNF-α, ⑫IL-6, ⑬TLR-4, ⑭ET-1, ⑮NO, ⑯MDA, ⑰SOD, ⑱WBSV, ⑲PSV, ⑳fibrinogen, ㉑CD62p, ㉒CD63, ㉓adverse response.

### Literature quality evaluation of included studies

**FIGURE 2:**
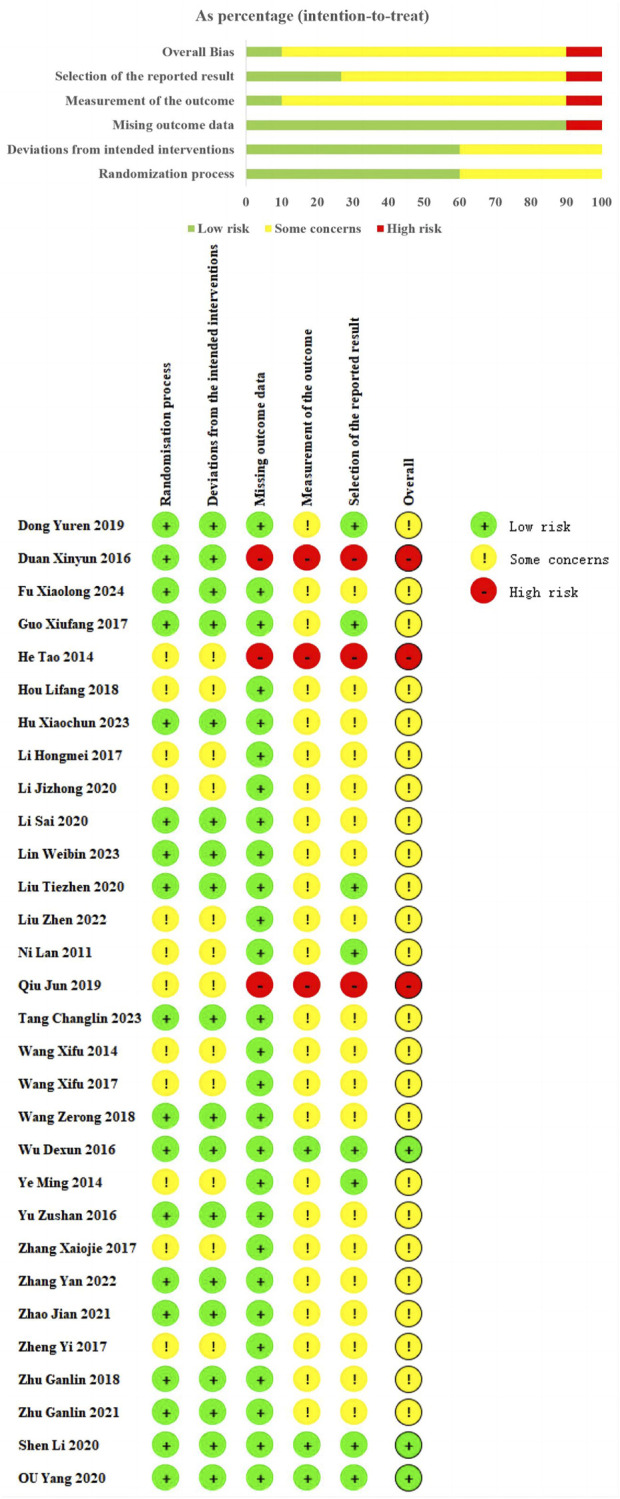
Risk of bias of included studies.

Among the 30 studies included, all used random sequence generation. However, 12 studies (Wang et al., 2017; Yi et al., 2017; Wang et al., 2013; Ye et al., 2014; Qiu, 2019; Liu et al., 2022; Zhang et al., 2017; Hongmei et al., 2017; Jizheng and Lu, 2020; He et al., 2014; Hou and Yunfeng, 2018; Lan et al., 2011) did not detail the randomization method, resulting in an “some concerns.” These missing details introduce potential risks of selection bias, which could affect the reliability of the reported outcomes. Eighteen studies provided clear descriptions of their randomization methods, including 15 studies (Shen L. et al., 2020; Ou et al., 2020; Wang, 2018; Zu-shan et al., 2016; Wu et al., 2016; Tang, 2023; Zhang et al., 2022; Hu Xiaochun et al., 2023; Guo, 2017; Duan and Jian-Hong, 2016; Dong et al., 2019; Li et al., 2020; Zhu and Wang, 2022; Fu, 2024; Lin et al., 2023) using random number tables, 2 study (Zhu and Wang, 2018; Zhao, 2021) using coin-toss method, 1 study (Liu et al., 2020) using drew lots method, and were evaluated as “low risk.” Regarding measurement of the outcome and mising outcome data. Three study (Shen L. et al., 2020; Ou et al., 2020; Wu et al., 2016) reported some details regarding the methods of allocation concealment, and rated as “low risk.” Three RCTs (Qiu, 2019; Duan and Jian-Hong, 2016; He et al., 2014) were rated as “high risk” due to incomplete data, which could introduce bias into the analysis. The remaining studies were classified as “some concerns” due to insufficient information on these aspects, raising concerns about the potential for unrecognized biases. Regarding the selection of reported results, we rated nine trials (Shen L. et al., 2020; Ou et al., 2020; Wu et al., 2016; Ye et al., 2014; Liu et al., 2020; Guo, 2017; Dong et al., 2019; He et al., 2014; Lan et al., 2011) as “low risk” because the adverse events or adverse reactions were mentioned in the results. However, for the remaining studies, the absence of reported safety events raises the possibility of selective reporting bias (Figure 2).

## Main efficacy outcomes

### MACEs

**FIGURE 3:**
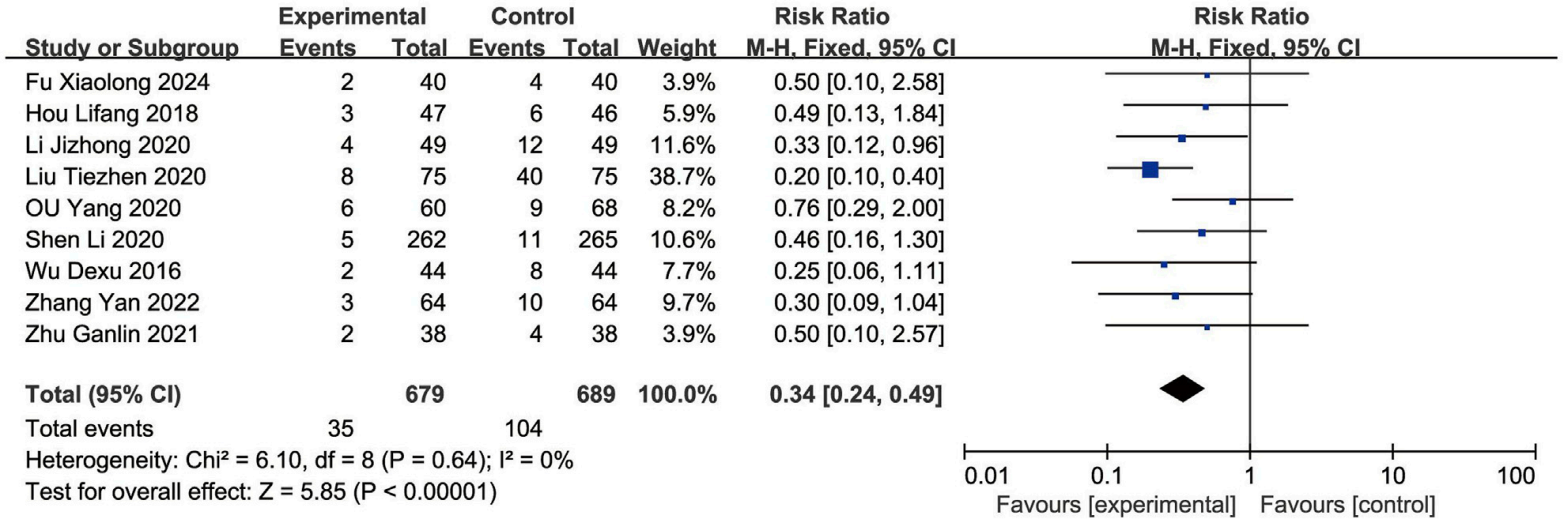
Forest plot of MACEs.

Nine RCTs (Shen L. et al., 2020; Ou et al., 2020; Wu et al., 2016; Liu et al., 2020; Zhang et al., 2022; Jizheng and Lu, 2020; Hou and Yunfeng, 2018; Zhu and Wang, 2022; Fu, 2024) reported the MACEs. As the heterogeneity was not significant (*p* = 0.64, *I* ^*2*^ = 0%), a fixed-effects model was used to analysis. The result showed that the occurrence of MACEs in the EG was significantly lower than in the CG, with the difference being statistically significant [RR = 0.34, (0.24–0.49), *p* < 0.05] (Figure 3).

## Secondary efficacy outcomes

### Myocardial injury

**FIGURE 4:**
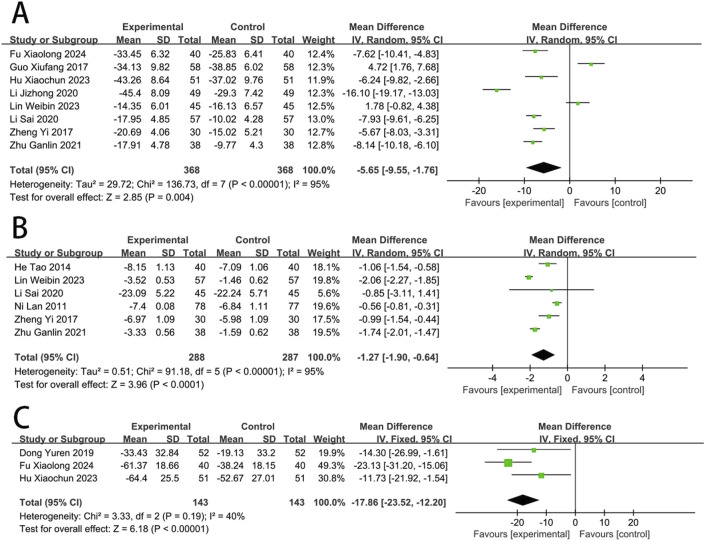
Forest plot of myocardial injury [**(A)** CK-MB; **(B)** cTnI; **(C)** LDH].

Eight RCTs (Yi et al., 2017; Zhu and Wang, 2018; Hu Xiaochun et al., 2023; Jizheng and Lu, 2020; Guo, 2017; Li et al., 2020; Fu, 2024; Lin et al., 2023) reported the CK-MB. The meta-analysis revealed a significant reduction in CK-MB concentrations in the EG compared to the CG [MD = −5.65, (−9.55 to −1.76), *p* < 0.05; *I* ^2^ = 95%, random-effects model]. Six RCTs (Yi et al., 2017; He et al., 2014; Li et al., 2020; Zhu and Wang, 2022; Lan et al., 2011; Lin et al., 2023) reported the cTnI. Meta-analysis showed that cTnI was significantly reduced in EG compared to CG [MD = −1.27, (−1.90 to −0.64), *p* < 0.05; *I* ^2^ = 95%]. Three RCTs (Hu Xiaochun et al., 2023; Dong et al., 2019; Fu, 2024) reported the LDH, and meta-analysis showed that the LDH was significantly reduced in EG compared to CG [MD = −17.86, (−23.52 to −12.20), *p* < 0.05; *I* ^2^ = 40%] (Figure 4).

### Cardiac function

**FIGURE 5:**
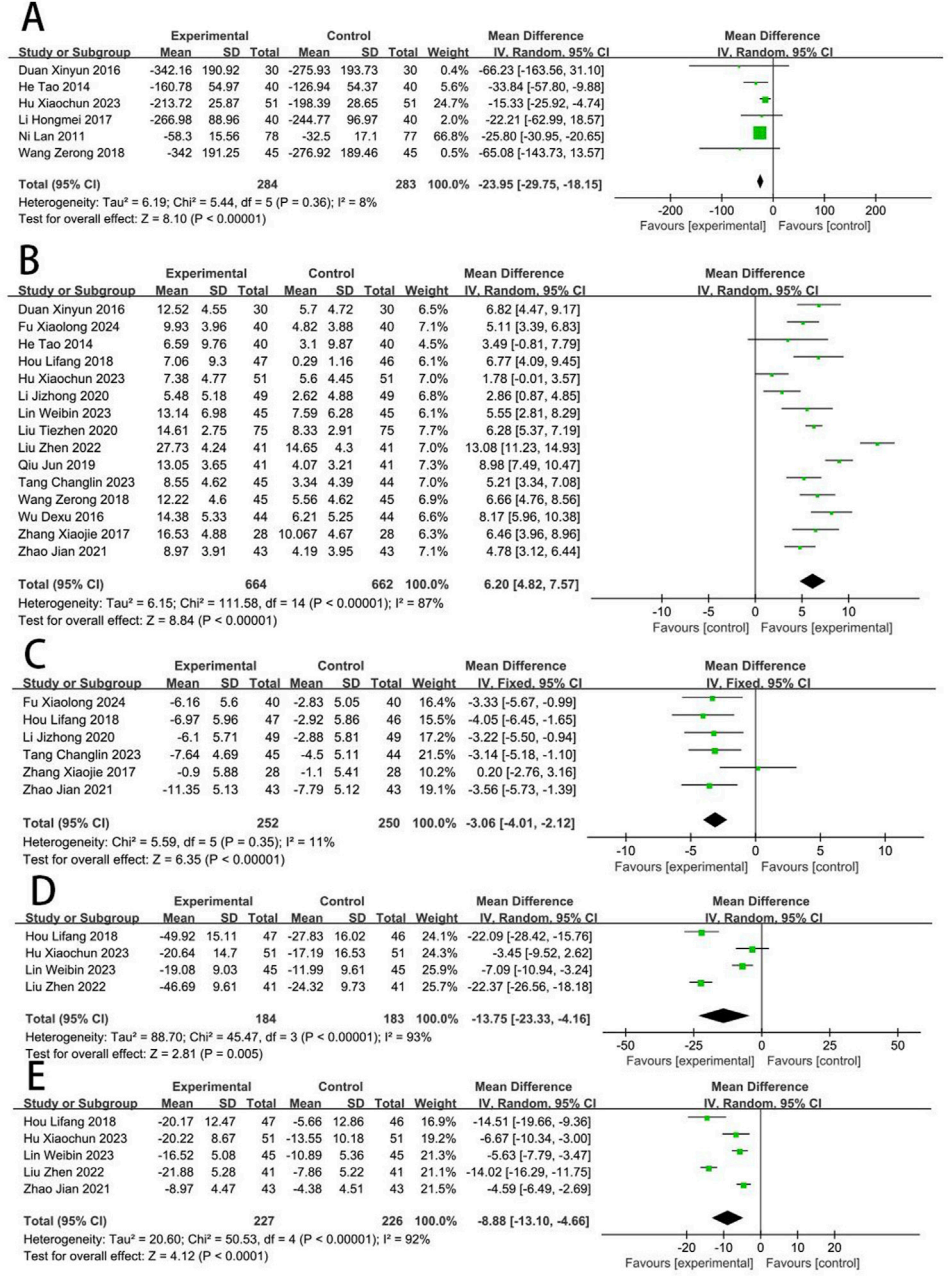
Forest plot of cardiac function [**(A)** BNP; **(B)** LVEF; **(C)** LVEDD; **(D)** LVEDV; **(E)** LVESV].

Six RCTs (Wang, 2018; Qiu, 2019; Hu Xiaochun et al., 2023; Hongmei et al., 2017; Duan and Jian-Hong, 2016; He et al., 2014; Lan et al., 2011) reported the NT-proBNP. The meta-analysis revealed that the NT-proBNP in the EG was significantly lower compared to the CG [MD = −24.36, (−28.87 to −19.86), *p* < *0.05*; *I* ^2^ = 8%]. Fifteen RCTs (Shen L. et al., 2020; Wang, 2018; Wu et al., 2016; Qiu, 2019; Tang, 2023; Liu et al., 2020; Liu et al., 2022; Zhang et al., 2017; Hu Xiaochun et al., 2023; Jizheng and Lu, 2020; Duan and Jian-Hong, 2016; He et al., 2014; Hou and Yunfeng, 2018; Fu, 2024; Zhao, 2021; Lin et al., 2023) reported the LVEF, and meta-analysis revealed that the LVEF in EG was significantly higher compared to the CG [MD = 6.2, (4.82–7.57), *p* < 0.05; *I* ^2^ = 87%]. Six RCTs (Tang, 2023; Zhang et al., 2017; Jizheng and Lu, 2020; Hou and Yunfeng, 2018; Fu, 2024; Zhao, 2021) reported the LVEDD, and revealed that the LVEDD in EG is significantly lower compared to the CG [MD = −3.06, (−4.01 to −2.12), *p* < 0.05; *I* ^2^ = 11%]. Four RCTs (Liu et al., 2022; Hu Xiaochun et al., 2023; Hou and Yunfeng, 2018; Lin et al., 2023) reported the LVEDV, and showed that the LVEDV in EG is significantly lower compared to the CG [MD = −13.75, (−23.33 to −4.16), *p* < 0.05; *I* ^2^ = 93%]. Five RCTs (Liu et al., 2022; Hu Xiaochun et al., 2023; Hou and Yunfeng, 2018; Zhao, 2021; Lin et al., 2023) reported the LVESV, and revealed that the LVESV in EG is significantly lower compared to the CG [MD = −8.88, (−13.10 to −4.66), *p* < 0.05; *I* ^2^ = 92%] (Figure 5).

### Inflammatory response

**FIGURE 6:**
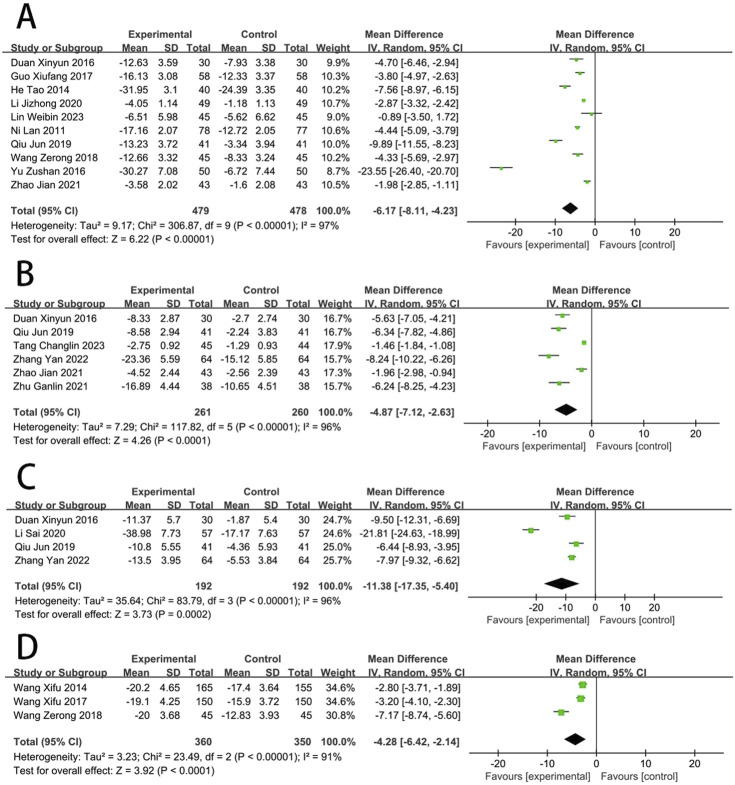
Forest plot of inflammatory response [**(A)** CRP; **(B)** TNF-α; **(C)** IL-6; **(D)** TLR-4].

Ten RCTs (Wang, 2018; Zu-shan et al., 2016; Qiu, 2019; Jizheng and Lu, 2020; Guo, 2017; Duan and Jian-Hong, 2016; He et al., 2014; Zhao, 2021; Lan et al., 2011; Lin et al., 2023) reported the CRP. The meta results suggested that SFI significantly improved CRP [MD = −6.17, (−8.11 to −4.23), *p* < 0.05; *I* ^2^ = 97%]. Six RCTs (Qiu, 2019; Tang, 2023; Zhang et al., 2022; Duan and Jian-Hong, 2016; Zhu and Wang, 2022; Zhao, 2021) reported the TNF-α amd meta results suggested that SFI significantly improved TNF-α [MD = −4.87, (−7.12 to −2.63), *p* < 0.05; *I* ^2^ = 96%]. Four RCTs (Qiu, 2019; Zhang et al., 2022; Duan and Jian-Hong, 2016; Li et al., 2020) reported the IL-6, and suggested that SFI significantly improved IL-6 [MD = −11.38, (−17.35 to −5.40), *p* < 0.05; *I* ^2^ = 96%]. Three RCTs (Wang, 2018; Wang et al., 2017; Wang et al., 2013) reported the TLR-4, and suggested that SFI significantly improved TLR-4 [MD = −4.28, (−6.42 to −2.14), *p* < 0.05; *I* ^2^ = 91%] (Figure 6).

### Oxidative stress

**FIGURE 7:**
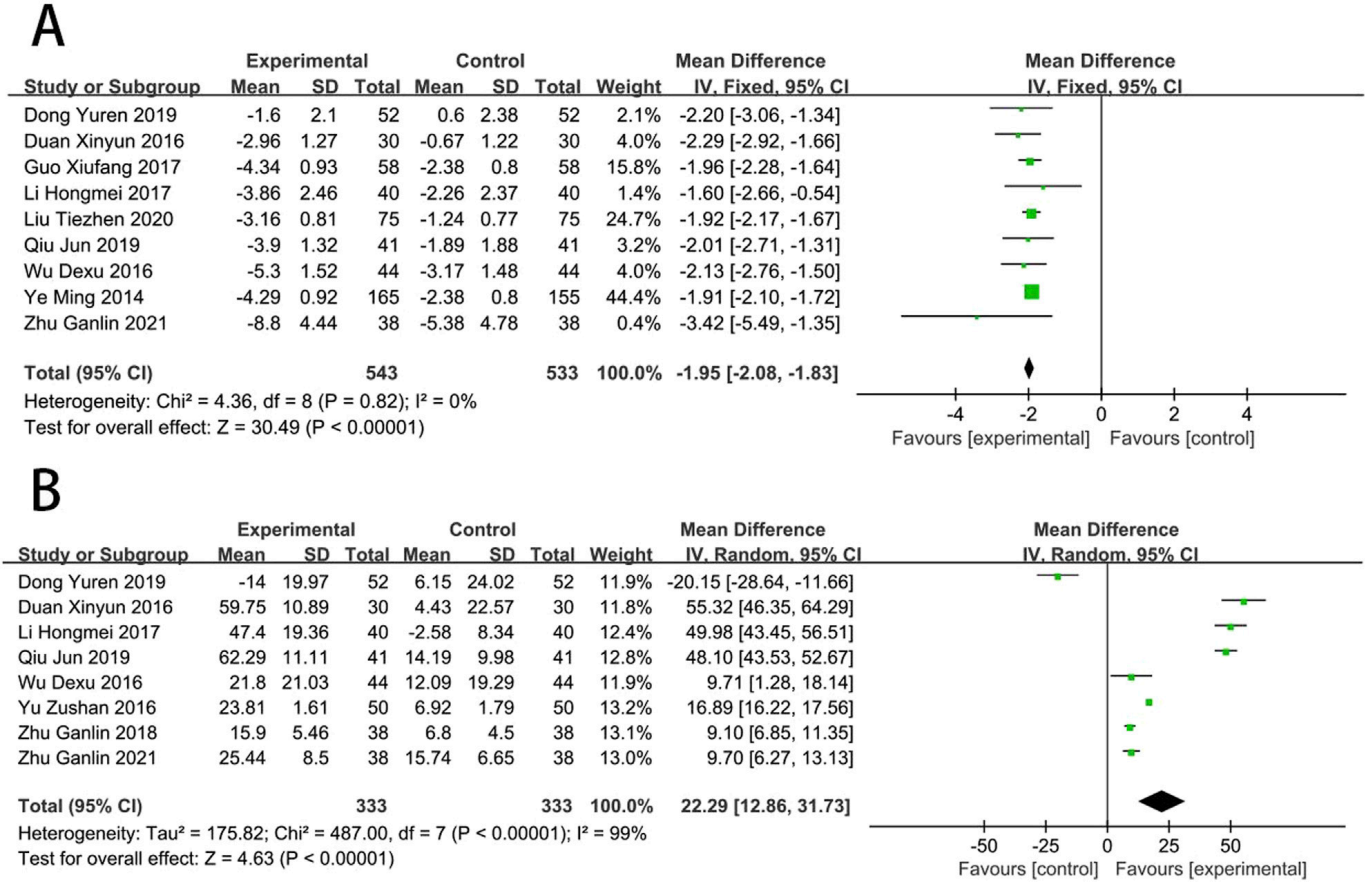
Forest plot of oxidative stress [**(A)** MDA; **(B)** SOD].

Nine RCTs (Wu et al., 2016; Ye et al., 2014; Qiu, 2019; Liu et al., 2020; Hongmei et al., 2017; Guo, 2017; Duan and Jian-Hong, 2016; Dong et al., 2019; Zhu and Wang, 2022) reported the MDA. Compared with CT, results suggested that SFI can significantly reduce the MDA [MD = −1.95, (−2.08 to −1.83), *p* < 0.05; *I* ^2^ = 0%]. Eight RCTs (Zu-shan et al., 2016; Wu et al., 2016; Qiu, 2019; Zhu and Wang, 2018; Hongmei et al., 2017; Duan and Jian-Hong, 2016; Dong et al., 2019; Zhu and Wang, 2022) reported the SOD. Compared with CT, results suggested that SFI can significantly increase the SOD [MD = 22.29, (12.86–31.73), *p* < 0.05; *I* ^2^ = 99%] (Figure 7).

### Vascular endothelial function

**FIGURE 8:**
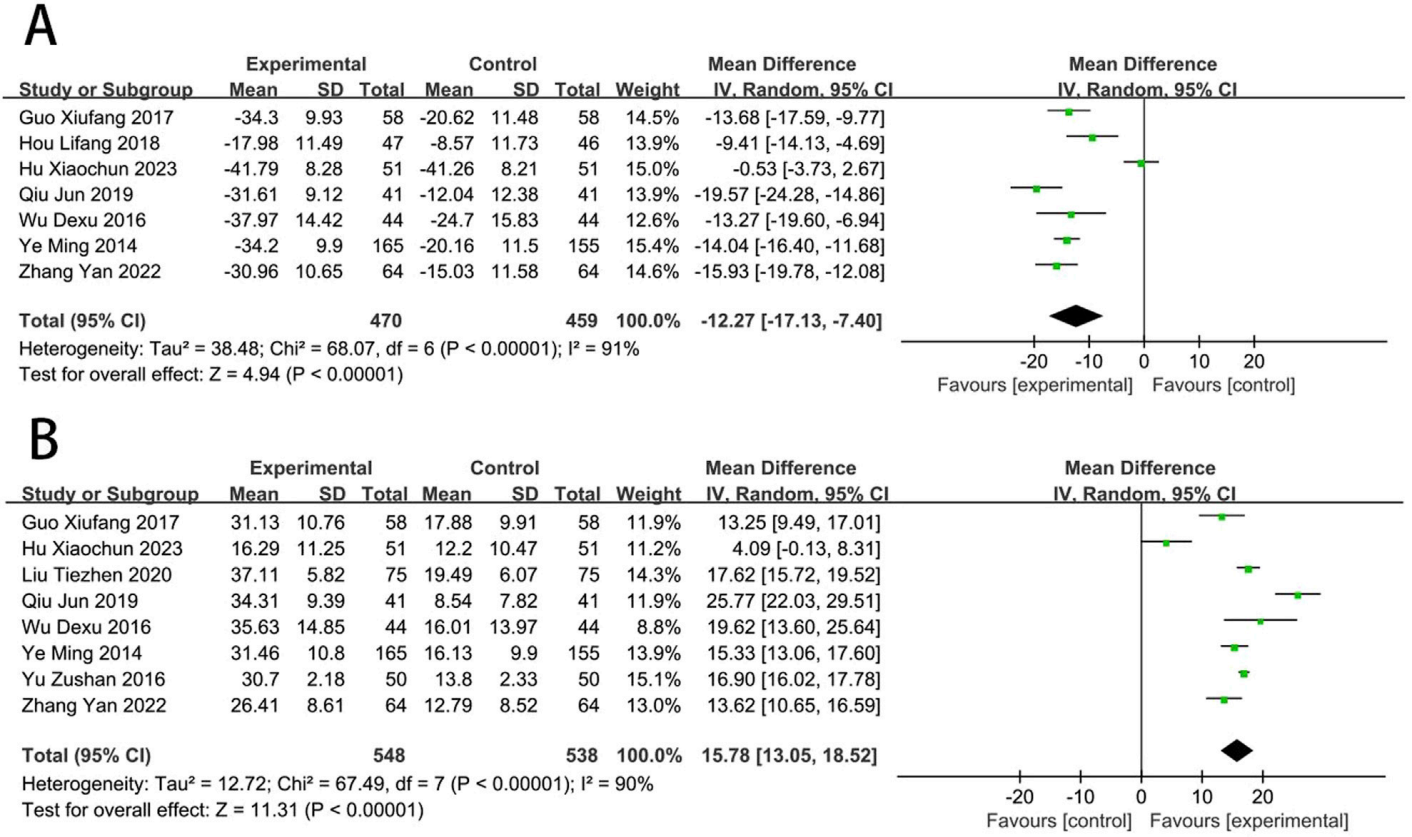
Forest plot of vascular endothelial function [**(A)** ET-1; **(B)** NO].

Seven RCTs (Wu et al., 2016; Ye et al., 2014; Qiu, 2019; Zhang et al., 2022; Hu Xiaochun et al., 2023; Guo, 2017; Hou and Yunfeng, 2018) reported the ET-1. The meta-analysis indicated that EG was more effective than CG on reducing the level of ET-1 [MD = −12.27, (−17.13 to −7.40), *p* < 0.05; *I* ^2^ = 91%]. Eight RCTs (Zu-shan et al., 2016; Wu et al., 2016; Ye et al., 2014; Qiu, 2019; Liu et al., 2020; Zhang et al., 2022; Hu Xiaochun et al., 2023; Guo, 2017) reported the NO, and meta-analysis indicated that EG was more effective than CG on increasing the NO [MD = 15.78, (13.05–18.52), *p* < 0.05; *I* ^2^ = 90%] (Figure 8).

### Hemorheological indicators

**FIGURE 9:**
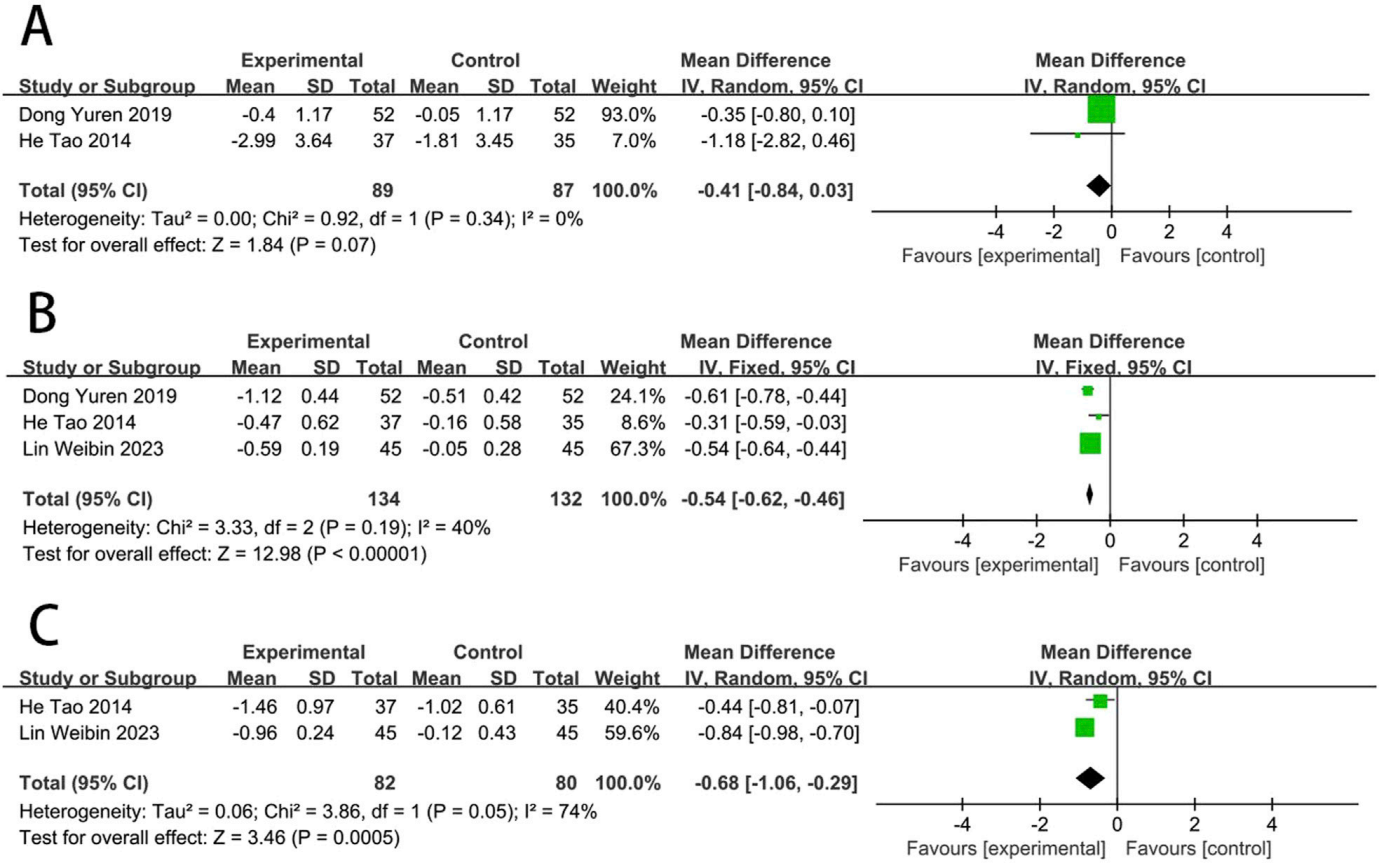
Forest plot of hemorheological function [**(A)** WBSV; **(B)** PSV; **(C)** fibrinogen].

Two RCTs (Dong et al., 2019; He et al., 2014) reported the WBSV. The results showed no statistically significant difference in WBSV between EG and CG [MD = −0.41, (−0.84 to 0.03), *p* > 0.05; *I* ^2^ = 0%]. Three RCTs (Dong et al., 2019; He et al., 2014; Lin et al., 2023) reported the PSV. The results showed that PSV levels improved better in EG than in CG [MD = −0.54, (−0.62 to −0.46), *p* < 0.05; *I* ^2^ = 40%]. Two RCTs (He et al., 2014; Lin et al., 2023) reported the fibrinogen. The results showed that fibrinogen levels improved better in EG than in CG [MD = −0.68, (−1.06 to −0.29), *p* < 0.05; *I* ^2^ = 74%] (Figure 9).

### Platelet function

**FIGURE 10:**
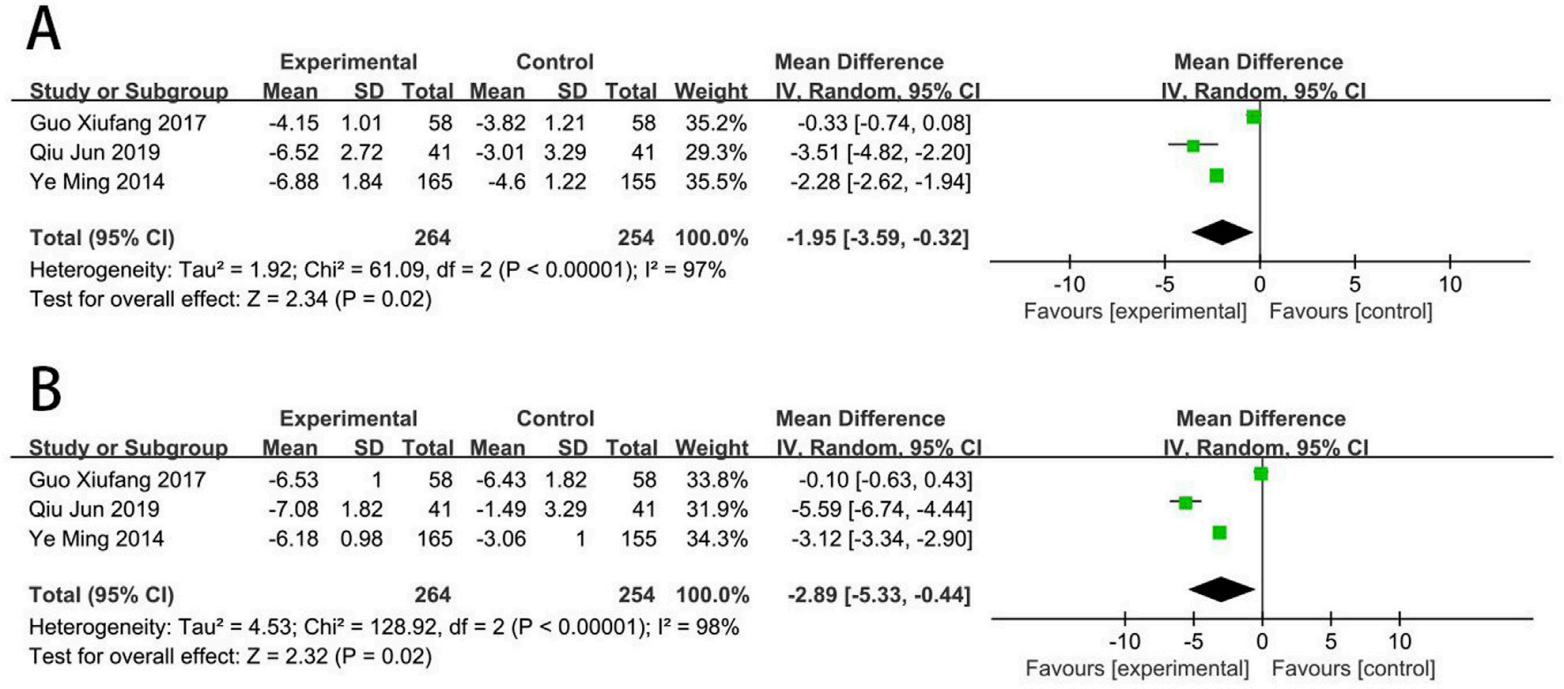
Forest plot of platelet function [**(A)** CD62p; **(B)** CD63].

Three RCTs (Ye et al., 2014; Qiu, 2019; Guo, 2017) reported the CD62p. Meta-analysis showed that EG was more effective than CG on reducing the level of CD62p [MD = −1.95, (−3.59 to −0.32), *p* < 0.05; *I* ^2^ = 97%]. Three RCTs (Ye et al., 2014; Qiu, 2019; Guo, 2017) reported the CD63. Meta-analysis showed that EG was more effective than CG on reducing the level of CD63 [MD = −2.89, (−5.33 to −0.44), *p* < 0.05; *I* ^2^ = 98%] (Figure 10).

## Safety outcomes

**FIGURE 11:**
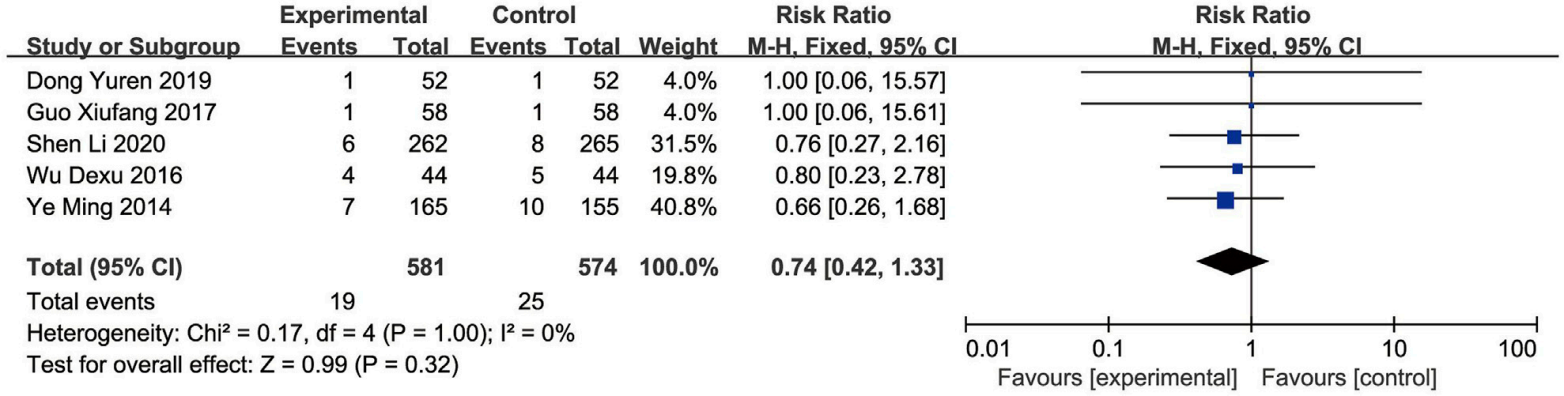
Forest plot of adverse events.

Nine RCTs (Shen L. et al., 2020; Ou et al., 2020; Wu et al., 2016; Ye et al., 2014; Liu et al., 2020; Guo, 2017; Dong et al., 2019; He et al., 2014; Lan et al., 2011) reported the adverse events. The Meta-analysis showed that the incidence of adverse events did not significantly differ between the EG and CG [RR = 0.74, 95% CI: 0.42 to 1.33, *p* = 0.32]. Given the non-significant heterogeneity (*p* = 1.00, I^2^ = 0%), a fixed-effects model was employed for the analysis (Figure 11). Across the included studies, four studies (Ou et al., 2020; Liu et al., 2020; He et al., 2014; Lan et al., 2011) found no adverse events in both the EG and CG. Other studies documented adverse events related to the AMI, including dizziness, rash, thrombocytopenia, hypotension, hemorrhage, and arrhythmia, with similar frequencies observed between the EG and CG. Overall, the reported adverse events were infrequent and comparable, indicating no major safety concerns related to SFI.

### Subgroup analysis

One subgroup analysis stratified by the follow-up duration (1-month, 3-month, and 6-month) indicated that SFI could decrease MACEs at 3-month checkpoint [RR = 0.38, (0.15–0.91), *p* < 0.05] and 6-month [RR = 0.25, (0.15–0.41), *p* < 0.05]. Nonetheless, there was no notable difference was observed at the 1-month [RR = 0.52, (0.25–1.09), *p* > 0.05] (Supplementary Figure 1).

Another subgroup analysis by the treatment duration of SFI (7-days or 14-days), indicated that SFI could decrease MACEs at 7-days checkpoint [RR = 0.49, (0.26–0.92), *p* < 0.05] and 14-days [RR = 0.29, (0.19–0.45), *p* < 0.05]. In addition, SFI could decrease CK-MB at 14-days checkpoint [MD = −7.35, (−11.39 to −3.32), *p* < 0.05]. However, there was no notable difference observed at the 7-days checkpoint of CK-MB [MD = 2.05, (−3.17–7.28), *p* > 0.05] (Supplementary Figure 2).

### Sensitivity analysis

When significant heterogeneity was observed (*I* ^*2*^ ≥ 50%) in outcomes such as CK-MB, cTnI, LVEF, LVEDV, LVESV, CRP, TNF-α, IL-6, ET-1, and NO, we conducted a sensitivity analysis. This analysis suggested that the variability could be attributed to differences in participant sample size, age, gender, and SFI intervention durations across trials. Excluding these studies substantially reduced the heterogeneity, with minimal impact on the overall results (Supplementary Table S4).

### Publication bias

Publication bias for LVEF and CRP was assessed using a funnel plot, as there were ten or more trials available. The results showed a symmetrical inverted funnel shape, indicating a low likelihood of publication bias for both LVEF and CRP (Supplementary Figure 3).

## Discussion

### Findings overview

To the best of our knowledge, this is the first study to systematically evaluate the efficacy and safety of SFI for AMI treatment. The findings reveal a substantial decrease in MACEs among AMI patients receiving SFI treatment. Additionally, SFI was found to mitigate myocardial injury, enhance cardiac function, reduce inflammatory responses and oxidative stress, and improve vascular endothelial, hemorheological, and platelet function. These effects likely contribute to the cardioprotective mechanism of action of SFI in AMI. Safety evaluations indicate that SFI does not increase the risk of adverse events, particularly bleeding. These findings highlight the efficacy and safety of SFI, demonstrating enhanced structural and functional outcomes in AMI patients and suggesting SFI as a promising treatment option for AMI.

### The mechanism of SFI for treating AMI

**FIGURE 12:**
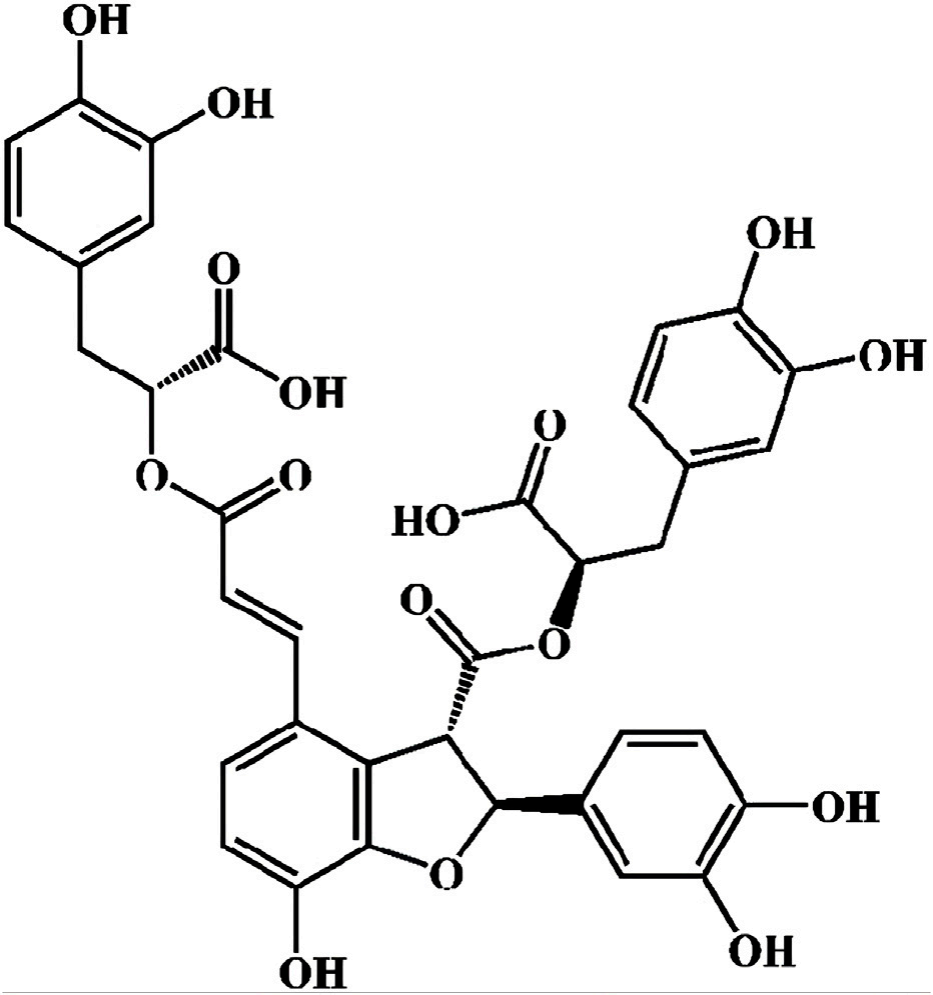
The molecular structural of Sal B.

The chemical metabolites of Danshen extract are primarily categorized into two groups: water-soluble metabolites and lipophilic phenanthraquinones. The principal lipophilic phenanthraquinones include Tanshinone I, Tanshinone IIA, and Tanshinone IIB. The major water-soluble metabolites comprise Danshensu, Rosmarinic Acid, Lithospermic Acid, Salvianolic Acid A, and Sal-B (Ho and Hong, 2011; Wang et al., 2019; Li ZM. et al., 2018). Sal-B, also referred to as lithospermic acid B, is a key bioactive metabolite found in the hydrophilic extracts of Salvia miltiorrhiza, with a molecular formula of C36H30O16 and a molecular weight of 718 (Figure 12). The SFI formula is predominantly composed of Sal-B, which has been recognized for its cardiovascular protective effects, particularly in mitigating oxidative stress, inflammation, and myocardial injury. Sal-B’s magnesium salt derivative, magnesium tanshinoate B (MTB), may offer enhanced therapeutic potential in treating AMI due to the physiological benefits of magnesium ions on cardiac muscle function (Xiao et al., 2020). Given that Sal-B constitutes 80% of the active metabolites in SFI and has been more extensively studied than other metabolites, this investigation primarily focuses on the pharmacological effects of Sal-B in AMI treatment.

Oxidative stress-induced injury plays a crucial role in causing severe and irreversible damage to cardiomyocytes in AMI. Sal-B exhibits potent antioxidant activity, effectively neutralizing free radicals and attenuating myocardial cell damage caused by oxidative stress (Xiao et al., 2020; Wu et al., 2009). The cardioprotective effects of Sal-B are mediated through the enhancement of SOD activity and the reduction of MDA production. Furthermore, Sal-B significantly protects bone marrow-derived endothelial progenitor cells from oxidative stress-induced injury by inhibiting the MKK3/6-p38, MAPK-ATF2, and ERK1/2 signaling pathways, thereby reducing intracellular reactive oxygen species (ROS) levels (Tang et al., 2014). Research conducted by Gao et al. demonstrated that Sal-B protects human umbilical vein endothelial cells (HUVECs) from oxidative stress, partially through the enhancement of autophagy via activation of the AMPK pathway and suppression of the mTOR pathway (Gao S. et al., 2019). Additionally, Lu et al. (2022) reported that Sal-B shields cardiomyocytes from ischemia/reperfusion (I/R)-induced oxidative stress both *in vitro* and *in vivo*, a process partially mediated by the TRIM8/GPX1 axis.

The inflammatory response plays a pivotal role in myocardial injury and remodeling following AMI. Sal-B has been demonstrated to inhibit the release of various inflammatory mediators, including CRP, TNF-α, IL-16, and TLR-4, primarily through the modulation of the TNF-α/NF-κB and TLR pathways. Researches conducted by Hu Y. et al. (2019) and Hu et al. (2020) revealed that Sal-B significantly attenuated AMI injury induced by subcutaneous isoproterenol (ISO) injection in a rat model. Sal-B treatment was observed to markedly reduce intracellular ROS production, inhibit NLRP3 inflammasome activation, and decrease apoptosis in H9C2 cardiomyocytes. Furthermore, Sal-B has been shown to promote the formation of anti-inflammatory M2 macrophages by regulating macrophage polarization, thereby mitigating myocardial damage caused by the inflammatory response (Li et al., 2021; Zou et al., 2022).

Cardiomyocyte apoptosis represents a key mechanism of myocardial injury following AMI. Sal-B has been found to reduce markers of myocardial damage, including CK-MB, cTnI, and LDH. The anti-apoptotic effects of Sal-B are mediated through the regulation of the Bcl-2/Bax expression ratio and the suppression of caspase activation (Wang et al., 2022; Chen HM. et al., 2017). Additionally, Sal-B exerts cardioprotective effects by activating the PI3K/Akt signaling pathway, thereby enhancing cell survival signals (Liu et al., 2007). Research conducted by Liu et al. (Liu et al., 2020) demonstrated that Sal-B significantly ameliorated myocardial I/R injury in a dose-dependent manner. This protective effect was characterized by a reduction in myocardial infarction size, decreased expression of myocardial injury markers, attenuated cell apoptosis, enhanced PI3K/Akt expression, and inhibition of high mobility group box 1 (HMGB1) expression.

Sal-B has been shown to exhibit antiplatelet and anticoagulant properties through multiple mechanisms. The metabolite reduces the expression of CD62p on the platelet surface and inhibits platelet activation induced by adenosine diphosphate (ADP), collagen, and thrombin (Chen et al., 2023). Moreover, Sal-B demonstrates anticoagulant effects by decreasing thrombin production and fibrin formation, thereby preventing blood clot formation. A study conducted by Liu et al. (Liu et al., 2014) elucidated two primary mechanisms underlying the antiplatelet effects of Sal-B: firstly, as a P2Y12 receptor antagonist, Sal-B significantly inhibits the interaction between ADP and the P2Y12 receptor; secondly, it inhibits phosphodiesterase (PDE) activity in platelets, preventing cyclic adenosine monophosphate (cAMP) degradation and thus suppressing platelet activation. Furthermore, Sal-B has been observed to reduce Ca^2+^ mobilization within platelets, further contributing to its antiplatelet aggregation effects (Song et al., 2017; Chen RC. et al., 2017).

Endothelial dysfunction has been identified as a crucial factor in the pathophysiology of AMI. Sal-B has been demonstrated to improve endothelial function through the promotion of endothelial cell survival and repair mechanisms. Extensive research has elucidated that Sal-B enhances the synthesis and release of NO, thereby improving vasodilation and reducing endothelial cell apoptosis (Ling et al., 2017). Furthermore, Sal-B exerts protective effects on endothelial cells by attenuating oxidative stress and inflammatory responses (Ren et al., 2016; Ko et al., 2020). A study conducted by Chen et al. (Chen et al., 2001) revealed that Sal-B primarily safeguards vascular endothelial cells through the concentration-dependent reduction of vascular cell adhesion molecule-1 (VCAM-1) and intercellular adhesion molecule-1 (ICAM-1) activity.

Pharmacological investigations have confirmed that hemodynamic alterations represent a primary etiological factor in thrombosis formation. Fibrinogen plays a pivotal role in platelet aggregation, a reduction in fibrinogen levels can lead to decreased thrombus formation. Sal-B has been shown to significantly lower fibrinogen and lipid peroxide levels, while concomitantly increasing high-density lipoprotein (HDL) concentrations. These effects contribute to improved blood viscosity and a reduction in the nitric oxide/endothelin (NO/ET) ratio (Dong et al., 2019; He et al., 2014; Lin et al., 2023). Moreover, Sal-B enhances vascular dilation and improves hemodynamics through the modulation of vascular smooth muscle cell function (He H. et al., 2008). Clinical studies have demonstrated that Sal-B administration significantly reduces blood viscosity, increases red blood cell deformability, and improves microcirculation in patients with AMI (He HB. et al., 2008).

Sal-B has been demonstrated to mitigate fibrosis and cardiac remodeling while promoting angiogenesis. In AMI rat models, Sal-B selectively inhibits MMP-9 activity, effectively augments left ventricular wall thickness, enhances cardiac contractility, and attenuates myocardial fibrosis (Ma et al., 2015; Sun et al., 2021; Luo et al., 2023). The metabolite inhibits type I collagen production in the LX-2 cell line under non-TGF-β2 stimulation and exerts anti-fibrotic effects through the inhibition of p38 and ERK signaling pathways (Luo et al., 2023; Gao H. et al., 2019; Li et al., 2020). Clinical studies have revealed that Sal-B significantly elevates serum VEGF levels in AMI patients, thereby promoting vascular endothelial cell proliferation and migration and facilitating new blood vessel formation (He HB. et al., 2008; Chen et al., 2022; Dhapare and Sakagami, 2018). Furthermore, Sal-B enhances hypoxia-induced angiogenesis and promotes myocardial repair through the activation of the HIF-1α signaling pathway (Yang et al., 2016; Wei et al., 2018).

### Clinical application suggestions

The findings of this study suggest that SFI may play a therapeutic role in the AMI population. Subgroup analysis further revealed that SFI significantly reduced MACEs at 3 and 6 months follow-up, while no significant reduction was observed at 1 month. These results indicate that SFI can potentially reduce mortality and the incidence of recurrent myocardial infarction, thereby improving the long-term prognosis of AMI patients. Additional subgroup analysis demonstrated SFI’s efficacy in reducing MACE at 7 and 14 days of treatment. However, SFI reduced CK-MB at 14 days, with no significant difference observed at 7 days. Based on these findings, a treatment duration of 2 weeks is recommended.

### Comparison with existing literature

Our findings contribute to the growing body of evidence on the clinical applications of SFI. Yang et al. (Yang et al., 2020) conducted a retrospective analysis using national health insurance data in China to assess the economic impact of SFI in coronary heart disease (CHD). The study demonstrated that SFI was associated with lower hospitalization costs and shorter hospital stays compared to other treatments, such as Danhong and alprostadil injections, highlighting its potential to reduce the financial burden on patients and healthcare systems. However, their focus on economic outcomes left a gap in clinical efficacy assessment. By integrating our RCT-based clinical findings with Yang et al.’s economic insights, we emphasize the dual value of SFI—not only as a clinically effective treatment for AMI but also as a cost-efficient therapy for broader cardiovascular care.

Similarly, Shen Y. et al. (2020) explored SFI’s therapeutic potential in a different clinical setting—diabetic nephropathy. Their meta-analysis revealed that SFI improved renal function, reduced oxidative stress, and lowered inflammatory markers, demonstrating its potential beyond cardiovascular conditions. These therapeutic effects align with the cardioprotective benefits observed in our study. However, both studies, including ours, face limitations such as variability in methodological quality, differences in treatment regimens, and a lack of long-term follow-up. These limitations underscore the need for larger, high-quality RCTs to validate salvianolate’s long-term efficacy and safety across different clinical populations and to explore optimal dosing strategies for both cardiovascular and non-cardiovascular applications.

### Implications for future research

This study highlights SFI as a promising alternative treatment option for AMI. Despite these encouraging results, several key areas warrant further investigation. While SFI has demonstrated notable effectiveness in reducing MACEs, determining the optimal dosage to ensure maximum efficacy and minimal side effects remains crucial. Future research should prioritize dosage optimization and conduct long-term follow-ups to evaluate the sustained efficacy and safety of SFI across diverse patient populations. The study also reveals SFI’s significant improvement of myocardial injury markers, cardiac function, and inflammatory responses. However, its precise mechanisms of action remain elucidated. Future studies should delve into the molecular and cellular mechanisms of SFI to enhance understanding of its role in AMI management and to identify potential biological pathways and targets.

## Advantages and limitations

This study presents several notable strengths. Firstly, a comprehensive search was conducted across multiple databases without language or time restrictions. Secondly, the involvement of two independent investigators in study selection, data extraction, and bias assessment minimized the potential for errors. Thirdly, the application of rigorous standards in assessing and reviewing eligible trials ensured methodological robustness and aimed to draw unbiased conclusions. Lastly, an in-depth discussion of the mechanism of Sal-B in AMI treatment was provided.

Nevertheless, certain limitations must be acknowledged. Our findings should be interpreted with caution due to the risk of bias identified in some included studies. In particular, the absence of clear randomization methods and incomplete outcome reporting in some studies raise serious concerns about selection and reporting biases. These limitations suggest that some of the included studies may not be sufficiently reliable for assessing the efficacy and safety of SFI. To address these concerns, we conducted sensitivity analyses by excluding high-risk studies and found that the main results remained consistent, indicating a certain degree of robustness. In addition, subgroup analyses based on study characteristics showed no significant differences, further supporting the stability of our findings. However, the quality of safety reporting across the studies remains a concern. Some studies reported no adverse events, which could reflect either genuine safety or incomplete monitoring, increasing the possibility of selective reporting bias. Moreover, we acknowledge that the overall quality of the evidence remains a concern, and the limitations of some included studies restrict the strength of our conclusions. Therefore, while our findings provide preliminary evidence for the therapeutic potential of SFI, future studies should employ rigorous RCT designs with clear randomization methods, transparent reporting, and comprehensive outcome documentation to ensure more reliable and conclusive assessments.

## Conclusion

This study suggests that SFI may be a promising alternative therapy for treating AMI without increasing the risk of adverse events. However, our findings may be limited by the quality of the existing studies. High-quality RCTs are needed to provide more robust evidence.

## Data Availability

The original contributions presented in the study are included in the article/Supplementary Material, further inquiries can be directed to the corresponding authors.

## Supplementary material

SUPPLEMENTARY FIGURE S1 Subgroup analysis stratified by follow-up time of MACEs.

SUPPLEMENTARY FIGURE S2 Subgroup analysis stratified by treatment time of SAI [**(A)** MACEs; **(B)** CK-MB].

SUPPLEMENTARY FIGURE S3 The publication bias [**(A)** LVEF; **(B)** CRP].
