# Supplementary figures and images for: Efficacy and safety of salvianolate injection in treating acute myocardial infarction: a meta-analysis and systematic literature review
Source: Front Pharmacol. 2024 Dec 17;15:1478558. doi: 10.3389/fphar.2024.1478558 (PMC11685132; doi:10.3389/fphar.2024.1478558)

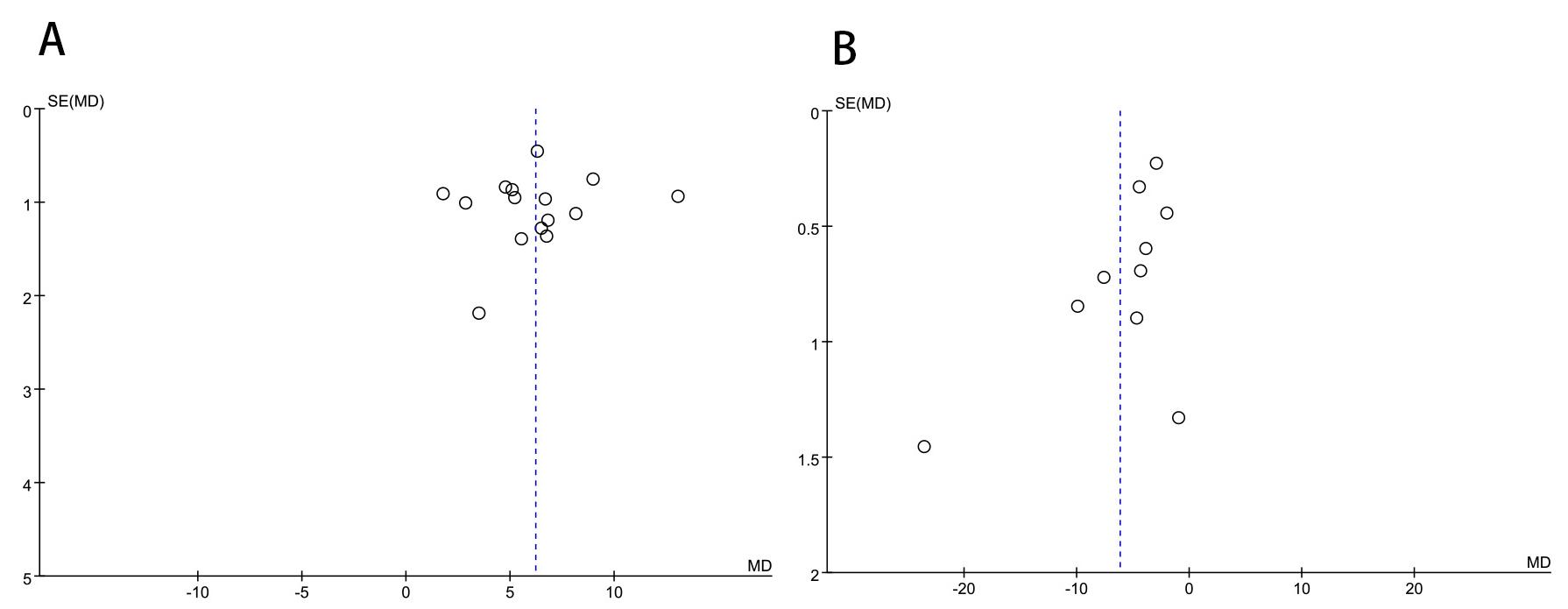

Supplement: Supplementary file 1 [file Image3.jpeg]

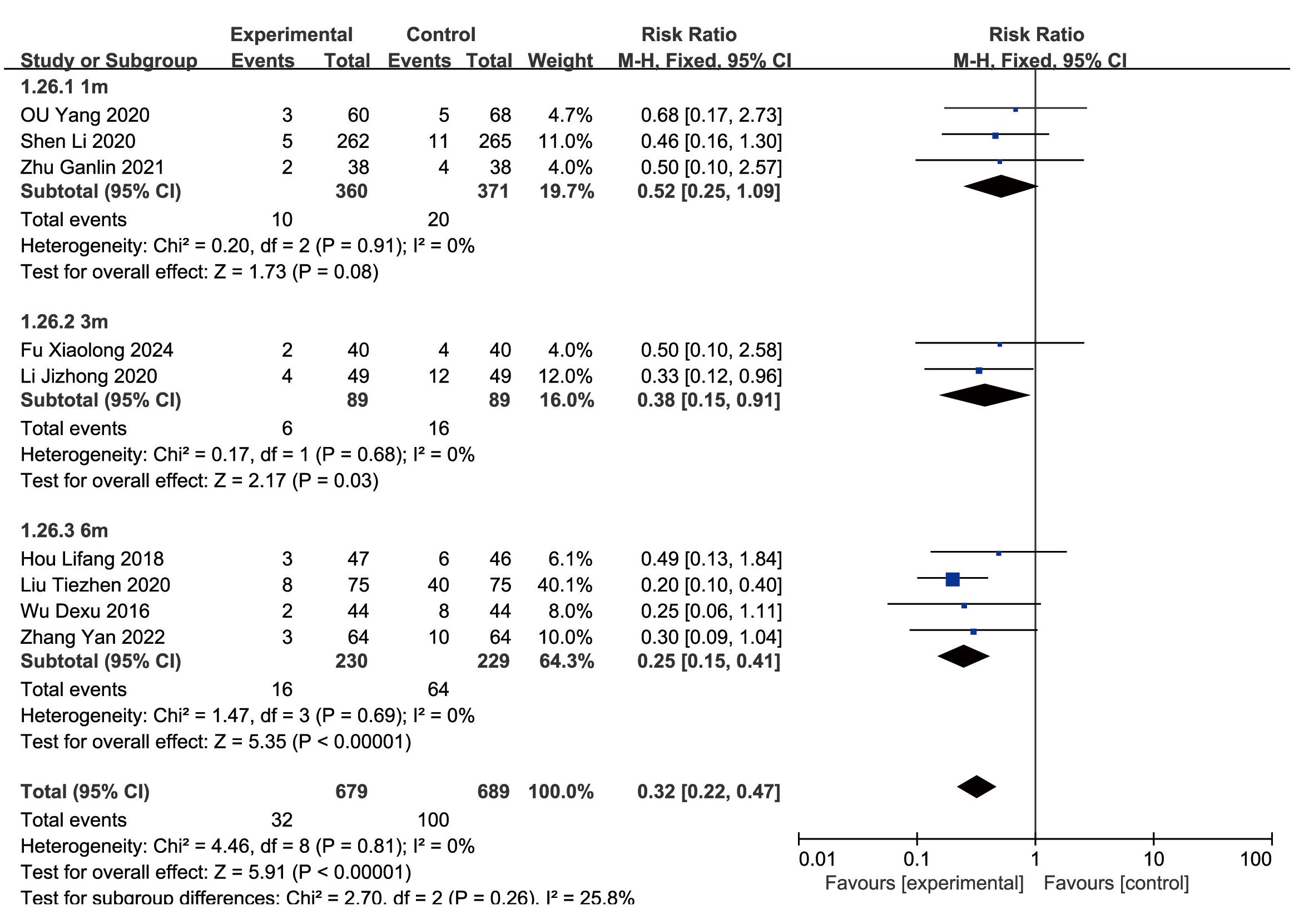

Supplement: Supplementary file 3 [file Image1.jpeg]

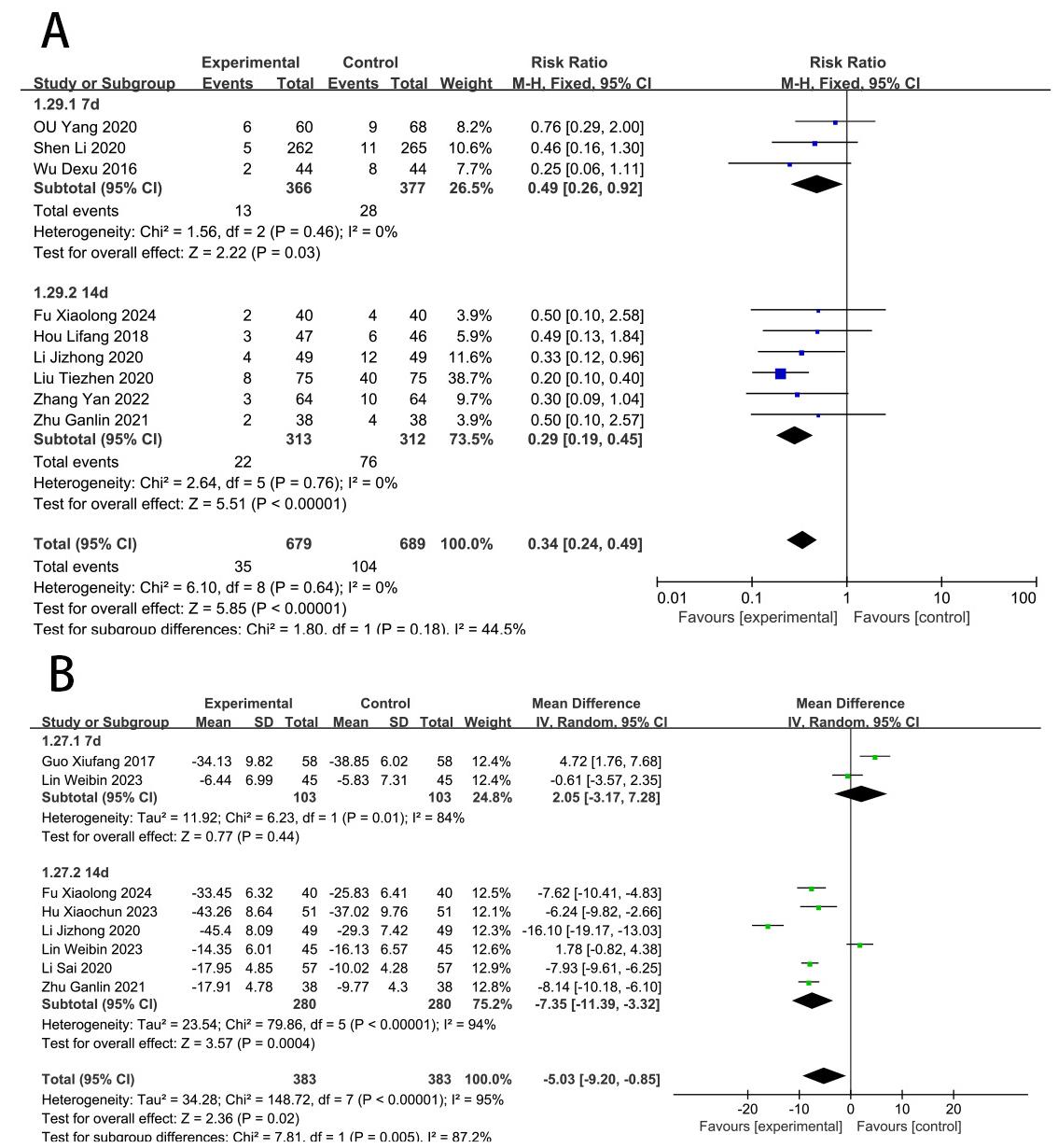

Supplement: Supplementary file 4 [file Image2.jpeg]
