# Supplementary material for: Efficacy and safety of salvianolate injection in treating acute myocardial infarction: a meta-analysis and systematic literature review
Source: Front Pharmacol. 2024 Dec 17;15:1478558. doi: 10.3389/fphar.2024.1478558 (PMC11685132; doi:10.3389/fphar.2024.1478558)
Supplement: Supplementary file 2 [file Table1.pdf]

Supplementary Table S1. Full Search Strategy and Results

Database (Search Date)	Search Terms	Filters	Number of results
Pubmed (1/7/2024)	#1 Acute myocardial infarction[mh] OR ST Elevation Myocardial Infarction[mh] OR Myocardial Infarction[mh] OR AMI[tiab] OR STEMI[tiab] OR ST Segment Elevation Myocardial Infarction*[tiab] OR ST Elevated Myocardial Infarction*[tiab]<br>#2 Salvianolate*[tiab] OR Salvianolic acids[tiab] OR Danshen polyphenolate salts[tiab]<br>#3 Randomized controlled trial[mh] OR RCT[tiab]<br>#4 #1 AND #2 AND #3	Language: English	35
Embase (1/7/2024)	#1 (Acute myocardial infarction).exp.<br>#2 (ST Elevation Myocardial Infarction).exp.<br>#3 (Myocardial Infarction).exp.<br>#4 (AMI).ti,ab.<br>#5 (STEMI).ti,ab.<br>#6 (ST Segment Elevation Myocardial Infarction).ti,ab.<br>#7 (ST Elevated Myocardial Infarction).ti,ab.<br>#8 #1 OR #2 OR #3 OR #4 OR #5 OR #6 OR #7<br>#9 (Salvianolate).ti,ab.<br>#10 (Salvianolic acids).ti,ab.<br>#11 (Danshen polyphenolate salts).ti,ab.<br>#12 #9 OR #10 OR #11<br>#13 (Randomized controlled trial).mp.#14 #8 AND #12 AND #13	Language: English	42
Web of science (1/7/2024)	#1 Topic:(Acute myocardial infarction OR ST Elevation Myocardial Infarction OR Myocardial Infarction OR AMI OR STEMI OR ST Segment Elevation Myocardial Infarction* OR ST Elevated Myocardial Infarction*)<br>Databases=SCI-EXPANDED, SSCI, A&HCI, CPCI-S, CPCI-SSH, BKCI-S, BKCI-SSH, ESCI, CCR-EXPANDED, IC Timespan= 1970-2024<br>#2 Topic:(Salvianolate* OR Salvianolic acids OR Danshen polyphenolate salts)<br>Databases=SCI-EXPANDED, SSCI, A&HCI, CPCI-S, CPCI-SSH, BKCI-S, BKCI-SSH, ESCI, CCR-EXPANDED, IC Timespan= 1970-2024<br>#3 Topic:(Randomized controlled trial)<br>Databases=SCI-EXPANDED, SSCI, A&HCI, CPCI-S, CPCI-SSH, BKCI-S, BKCI-SSH, ESCI, CCR-EXPANDED, IC Timespan= 1970-2024<br>#4 #1 AND #2 AND #3	Language: English	38
Cochrane Library (1/7/2024)	“Acute myocardial infarction” OR “ST Elevation Myocardial Infarction” OR “Myocardial Infarction” OR “AMI” OR “STEMI” OR “ST Segment Elevation Myocardial Infarction*” OR “ST Elevated Myocardial Infarction*” in Title Abstract Keyword AND “Salvianolate” OR “Salvianolic acids” OR	Language: English	9

	"Danshen polyphenolate salts" in Title Abstract Keyword AND "Randomized controlled trial" in Title Abstract Keyword - (Word variations have been searched)		
Chinese National Knowledge Infrastructure (1/7/2024)	("acute myocardial infarction" OR "AMI" OR "st elevated myocardial infarction" OR "STEMI") AND ("Salvianolate" OR "Salvianolic acids" OR "Danshen polyphenolate salts") AND ("randomized controlled trial" OR "RCT")	Language: Chinese	85
Wanfang (1/7/2024)	("acute myocardial infarction" OR "AMI" OR "st elevated myocardial infarction" OR "STEMI") AND ("Salvianolate" OR "Salvianolic acids" OR "Danshen polyphenolate salts") AND ("randomized controlled trial" OR "RCT")	Language: Chinese	76
VIP (1/7/2024)	("acute myocardial infarction" OR "AMI" OR "st elevated myocardial infarction" OR "STEMI") AND ("Salvianolate" OR "Salvianolic acids" OR "Danshen polyphenolate salts") AND ("randomized controlled trial" OR "RCT")	Language: Chinese	40

Supplementary Table S2. The Exclusion Studies and Reasons

**Reason for Exclusion**	**Study Name**	**Reference**
non-RCT study design	Ding L et al 2016	Salvianolic acid B protects against myocardial damage caused by nanocarrier TiO2; and synergistic anti-breast carcinoma effect with curcumin via codelivery system of folic acid-targeted and polyethylene glycol-modified TiO2 nanoparticles. Int J Nanomedicine. 2016, 2;11:5709-5727.
non-RCT study design	Yue QX et al 2012	Proteomic studies on protective effects of salvianolic acids, notoginsengnosides and combination of salvianolic acids and notoginsengnosides against cardiac ischemic-reperfusion injury. J Ethnopharmacol. 2012, 1;141(2):659-67.
non-RCT study design	Mao Q et al 2024	Exploring the Mechanism of Salvianolic Acid B against Myocardial Ischemia-Reperfusion Injury Based on Network Pharmacology. Pharmaceuticals (Basel). 2024,28;17(3):309.
non-RCT study design	Liu H et al 2020	Salvianolic acid B protects against myocardial ischaemia-reperfusion injury in rats via inhibiting high mobility group box 1 protein expression through the PI3K/Akt signalling pathway. Naunyn Schmiedebergs Arch Pharmacol. 2020,393(8):1527-1539.
non-RCT study design	Chen R et al 2021	An injectable peptide hydrogel with excellent self-healing ability to continuously release salvianolic acid B for myocardial infarction. Biomaterials. 2021,274:120855.
non-RCT study design	Li Y et al 2019	Cardioprotection of salvianolic acid B and ginsenoside Rg1 combination on subacute myocardial infarction and the underlying mechanism. Phytomedicine. 2019,57:255-261.
inaccurate data	Tian Yl et al 2011	Protective effects of salvianolate on ischemic myocardium and hemodynamics in patients with acute myocardial infarction [J]. Journal of Medical Informatics, 2011,34(22):63-65.
inaccurate data	Huang MJ et al 2019	Observation of salvianolate combined with Shengmai injection on ST segment regression and bleeding after STEMI stent implantation [J]. Popular Science and Technology, 2019,22(06):92-94.
inaccurate data	Ci LJ et al 2019	Effect of salvianolate combined with antiplatelet agents on serum inflammatory factors in acute myocardial infarction [J]. Journal of Aerospace Medicine,2019,30(10):1255-1257.
ineligible interventions	Han ZL 2018	Tirofiban, salvianolate and trimetazidine in treatment of STEMI after PCI [J]. Clinical Practice of Integrated Chinese and Western Medicine, 2018, 18 (12): 14-16.
ineligible interventions	Zhang ZP et al 2017	Salvianolate interferes with microRNA and target genes in patients with non-ST segment elevation myocardial infarction [J]. Chinese journal of experimental formulas of Chinese medicine, 2017, 23 (19) : 17-22.
ineligible interventions	Huang J et al 2017	Protective effect of salvia miltiorrhiza polyphenolate on nephropathy after emergency percutaneous coronary intervention [J]. Chinese Journal of Interventional Cardiology,2017,25(01):35-41.

Supplementary Table S3. The composition of the prescriptions

Study	Formulation	Source	Physical Properties	Specification	Main compound	Quality control reported	Chemical analysis
Dong Yuren 2019	Salvianolate 200 mg + 5% G.S. 250 mL	Shanghai Green Valley Life Garden Pharmaceutical Co., LTD	Lyophilized Powder	50mg	Sal-B 40mg	Prepared according to National Drug Standards of China Food and Drug Administration (National Drug Approval Number: Z20050247)	Sal-B, Lithospermic acid and Rosmarinic acid
Duan Xinyun 2016	Salvianolate 200 mg + 0.9% N.S. 250 mL	Shanghai Green Valley Life Garden Pharmaceutical Co., LTD	Lyophilized Powder	200mg	Sal-B 160mg	Prepared according to National Drug Standards of China Food and Drug Administration (National Drug Approval Number: Z20050249)	Sal-B, Lithospermic acid and Rosmarinic acid
Fu Xiaolong 2024	Salvianolate 150 mg + 0.9% N.S. 250 mL	Shanghai Green Valley Life Garden Pharmaceutical Co., LTD	Lyophilized Powder	50mg	Sal-B 40mg	Prepared according to National Drug Standards of China Food and Drug Administration (National Drug Approval Number: Z20050247)	Sal-B, Lithospermic acid and Rosmarinic acid
Guo Xiufang 2017	Salvianolate 200 mg + 5% G.S. 250 mL	Shanghai Green Valley Life Garden Pharmaceutical Co., LTD	Lyophilized Powder	200mg	Sal-B 160mg	Prepared according to National Drug Standards of China Food and Drug Administration (National Drug Approval Number: Z20050249)	Sal-B, Lithospermic acid and Rosmarinic acid
He Tao 2014	Salvianolate 200 mg + 5% G.S. 250 mL	Shanghai Green Valley Life Garden Pharmaceutical Co., LTD	Lyophilized Powder	200mg	Sal-B 160mg	Prepared according to National Drug Standards of China Food and Drug Administration (National Drug Approval Number: Z20050249)	Sal-B, Lithospermic acid and Rosmarinic acid
Hou Lifang 2018	Salvianolate 150 mg + 0.9% N.S. 250 mL	Shanghai Green Valley Life Garden Pharmaceutical Co., LTD	Lyophilized Powder	50mg	Sal-B 40mg	Prepared according to National Drug Standards of China Food and Drug Administration (National Drug Approval Number: Z20050247)	Sal-B, Lithospermic acid and Rosmarinic acid
Hu Xiaochun 2023	Salvianolate 150 mg + 0.9% N.S. 250 mL	Shanghai Green Valley Life Garden Pharmaceutical Co., LTD	Lyophilized Powder	50mg	Sal-B 40mg	Prepared according to National Drug Standards of China Food and Drug Administration (National Drug Approval Number: Z20050247)	Sal-B, Lithospermic acid and Rosmarinic acid
Li Hongmei 2017	Salvianolate 200 mg + 5% G.S. 250 mL	Shanghai Green Valley Life Garden Pharmaceutical Co., LTD	Lyophilized Powder	200mg	Sal-B 160mg	Prepared according to National Drug Standards of China Food and Drug Administration (National Drug Approval Number: Z20050249)	Sal-B, Lithospermic acid and Rosmarinic acid
Li Jizhong 2020	Salvianolate 200 mg + 5% G.S. 250 mL	Shanghai Green Valley Life Garden Pharmaceutical Co., LTD	Lyophilized Powder	200mg	Sal-B 160mg	Prepared according to National Drug Standards of China Food and Drug Administration (National Drug Approval Number: Z20050249)	Sal-B, Lithospermic acid and Rosmarinic acid
Li Sai 2020	Salvianolate 200 mg + 5% G.S. 250 mL	Shanghai Green Valley Life Garden Pharmaceutical Co., LTD	Lyophilized Powder	100mg	Sal-B 80mg	Prepared according to National Drug Standards of China Food and Drug Administration (National Drug Approval Number: Z20050248)	Sal-B, Lithospermic acid and Rosmarinic acid
Lin Weibin 2023	Salvianolate 200 mg + 0.9% N.S. 250 mL	Shanghai Green Valley Life Garden Pharmaceutical Co., LTD	Lyophilized Powder	200mg	Sal-B 160mg	Prepared according to National Drug Standards of China Food and Drug Administration (National Drug Approval Number: Z20050249)	Sal-B, Lithospermic acid and Rosmarinic acid

Liu Tiezhen 2020	Salvianolate 200 mg + 5% G.S. 250 mL	Shanghai Green Valley Life Garden Pharmaceutical Co., LTD	Lyophilized Powder	50mg	Sal-B 40mg	Prepared according to National Drug Standards of China Food and Drug Administration (National Drug Approval Number: Z20050247)	Sal-B, Lithospermic acid and Rosmarinic acid
Liu Zhen 2022	Salvianolate 200 mg + 0.9% N.S. 250 mL	Shanghai Green Valley Life Garden Pharmaceutical Co., LTD	Lyophilized Powder	100mg	Sal-B 80mg	Prepared according to National Drug Standards of China Food and Drug Administration (National Drug Approval Number: Z20050248)	Sal-B, Lithospermic acid and Rosmarinic acid
Ni Lan 2011	Salvianolate 200 mg + 5% G.S. 250 mL	Shanghai Green Valley Life Garden Pharmaceutical Co., LTD	Lyophilized Powder	200mg	Sal-B 160mg	Prepared according to National Drug Standards of China Food and Drug Administration (National Drug Approval Number: Z20050249)	Sal-B, Lithospermic acid and Rosmarinic acid
Qiu Jun 2019	Salvianolate 200 mg + 0.9% N.S. 250 mL	Shanghai Green Valley Life Garden Pharmaceutical Co., LTD	Lyophilized Powder	200mg	Sal-B 160mg	Prepared according to National Drug Standards of China Food and Drug Administration (National Drug Approval Number: Z20050249)	Sal-B, Lithospermic acid and Rosmarinic acid
Tang Changlin 2023	Salvianolate 200 mg + 5% G.S. 250 mL	Shanghai Green Valley Life Garden Pharmaceutical Co., LTD	Lyophilized Powder	200mg	Sal-B 160mg	Prepared according to National Drug Standards of China Food and Drug Administration (National Drug Approval Number: Z20050249)	Sal-B, Lithospermic acid and Rosmarinic acid
Wang Xifu 2014	Salvianolate 400 mg + 5% G.S. 250 mL	Shanghai Green Valley Life Garden Pharmaceutical Co., LTD	Lyophilized Powder	200mg	Sal-B 160mg	Prepared according to National Drug Standards of China Food and Drug Administration (National Drug Approval Number: Z20050249)	Sal-B, Lithospermic acid and Rosmarinic acid
Wang Xifu 2017	Salvianolate 200 mg + 5% G.S. 250 mL	Shanghai Green Valley Life Garden Pharmaceutical Co., LTD	Lyophilized Powder	200mg	Sal-B 160mg	Prepared according to National Drug Standards of China Food and Drug Administration (National Drug Approval Number: Z20050249)	Sal-B, Lithospermic acid and Rosmarinic acid
Wang Zerong 2018	Salvianolate 200 mg + 5% G.S. 250 mL	Shanghai Green Valley Life Garden Pharmaceutical Co., LTD	Lyophilized Powder	50mg	Sal-B 40mg	Prepared according to National Drug Standards of China Food and Drug Administration (National Drug Approval Number: Z20050247)	Sal-B, Lithospermic acid and Rosmarinic acid
Wu Dexun 2016	Salvianolate 200 mg + 0.9% N.S. 250 mL	Shanghai Green Valley Life Garden Pharmaceutical Co., LTD	Lyophilized Powder	200mg	Sal-B 160mg	Prepared according to National Drug Standards of China Food and Drug Administration (National Drug Approval Number: Z20050249)	Sal-B, Lithospermic acid and Rosmarinic acid
Ye Ming 2014	Salvianolate 400 mg + 0.9% N.S. 250 mL	Shanghai Green Valley Life Garden Pharmaceutical Co., LTD	Lyophilized Powder	200mg	Sal-B 160mg	Prepared according to National Drug Standards of China Food and Drug Administration (National Drug Approval Number: Z20050249)	Sal-B, Lithospermic acid and Rosmarinic acid
Yu Zushan 2016	Salvianolate 200 mg + 0.9% N.S. 250 mL	Shanghai Green Valley Life Garden Pharmaceutical Co., LTD	Lyophilized Powder	200mg	Sal-B 160mg	Prepared according to National Drug Standards of China Food and Drug Administration (National Drug Approval Number: Z20050249)	Sal-B, Lithospermic acid and Rosmarinic acid
Zhang Xaiojie 2017	Salvianolate 100 mg + 0.9% N.S. 100 mL	Shanghai Green Valley Life Garden Pharmaceutical Co., LTD	Lyophilized Powder	100mg	Sal-B 80mg	Prepared according to National Drug Standards of China Food and Drug Administration (National Drug Approval Number: Z20050248)	Sal-B, Lithospermic acid and Rosmarinic acid
Zhang Yan 2022	Salvianolate 200 mg + 5% G.S. 250 mL	Shanghai Green Valley Life Garden Pharmaceutical Co., LTD	Lyophilized Powder	200mg	Sal-B 160mg	Prepared according to National Drug Standards of China Food and Drug Administration (National Drug Approval Number: Z20050249)	Sal-B, Lithospermic acid and Rosmarinic acid

Zhao Jian 2021	Salvianolate 200 mg + 5% G.S. 250 mL	Shanghai Green Valley Life Garden Pharmaceutical Co., LTD	Lyophilized Powder	200mg	Sal-B 160mg	Prepared according to National Drug Standards of China Food and Drug Administration (National Drug Approval Number: Z20050249)	Sal-B, Lithospermic acid and Rosmarinic acid
Zheng Yi 2017	Salvianolate 200 mg + 5% G.S. 250 mL	Shanghai Green Valley Life Garden Pharmaceutical Co., LTD	Lyophilized Powder	200mg	Sal-B 160mg	Prepared according to National Drug Standards of China Food and Drug Administration (National Drug Approval Number: Z20050249)	Sal-B, Lithospermic acid and Rosmarinic acid
Zhu Ganlin 2018	Salvianolate 200 mg + 0.9% N.S. 100 mL	Shanghai Green Valley Life Garden Pharmaceutical Co., LTD	Lyophilized Powder	200mg	Sal-B 160mg	Prepared according to National Drug Standards of China Food and Drug Administration (National Drug Approval Number: Z20050249)	Sal-B, Lithospermic acid and Rosmarinic acid
Zhu Ganlin 2021	Salvianolate 200 mg + 5% G.S. 250 mL	Shanghai Green Valley Life Garden Pharmaceutical Co., LTD	Lyophilized Powder	200mg	Sal-B 160mg	Prepared according to National Drug Standards of China Food and Drug Administration (National Drug Approval Number: Z20050249)	Sal-B, Lithospermic acid and Rosmarinic acid
Shen Li 2020	Salvianolate 200 mg + 0.9% N.S. 100 mL	Shanghai Green Valley Life Garden Pharmaceutical Co., LTD	Lyophilized Powder	200mg	Sal-B 160mg	Prepared according to National Drug Standards of China Food and Drug Administration (National Drug Approval Number: Z20050249)	Sal-B, Lithospermic acid and Rosmarinic acid
OU Yang 2020	Salvianolate 200 mg + 0.9% N.S. 100 mL	Shanghai Green Valley Life Garden Pharmaceutical Co., LTD	Lyophilized Powder	200mg	Sal-B 160mg	Prepared according to National Drug Standards of China Food and Drug Administration (National Drug Approval Number: Z20050249)	Sal-B, Lithospermic acid and Rosmarinic acid

Supplementary Table S4. The results of sensitivity analysis

Outcome	Study removed [first author(year)]	I^2-value	OR/MD [95% CI], *P*-value
CK-MB	Guo Xiufang 2017	93%	-7.11[-10.51,-3.70], P<0.05
cTnI	Ni Lan 2011	83%	-1.49[-1.95,-1.04], P<0.05
LVEF	Liu Zhen 2022	77%	5.70[4.63,6.76], P<0.05
LVEDV	Liu Zhen 2022	90%	-10.74[-20.64,—0.84], P<0.05
LVESV	Liu Zhen 2022	77%	-7.06[-10.03,-4.09], P<0.05
CRP	Yu Zushan 2016	93%	-4.51[-5.83,-3.19], P<0.05
TNF-α	Tang Changlin 2023	92%	-5.61[-7.95,-3.27], P<0.05
IL-6	Li Sai 2020	23%	-7.90[-9.24,-6.56], P<0.05
ET-1	Hu Xiaochun 2023	49%	-14.37[-16.69,-12.05], P<0.05
NO	Hu Xiaochun 2023	82%	17.16[15.00,19.23], P<0.05
